# Cyclodextrin-Mediated Enantiomeric Separation of Idelalisib: A Validated Capillary Electrophoresis and NMR Study

**DOI:** 10.3390/ijms27136036

**Published:** 2026-07-05

**Authors:** Erzsébet Várnagy, Balázs István Urbán, Mátyás Sári, Balázs Volk, Gyula Simig, Krisztina Németh, Milo Malanga, Ida Fejős, Szabolcs Béni

**Affiliations:** 1 Department of Pharmacognosy, Semmelweis University, Üllői út 26, H-1085 Budapest, Hungary; varnagy.erzsebet@phd.semmelweis.hu; 2 Center for Pharmacology and Drug Research & Development, Semmelweis University, Üllői út 26, H-1085 Budapest, Hungary; 3 Integrative Health and Environmental Analysis Research Laboratory, Department of Analytical Chemistry, Institute of Chemistry, ELTE Eötvös Loránd University, Pázmány Péter Sétány 1/A, H-1117 Budapest, Hungary; urbanbalazsistvan@gmail.com (B.I.U.); sarimatyas03@gmail.com (M.S.); 4 Directorate of Drug Substance Development, Egis Pharmaceuticals Plc., P.O. Box 100, H-1475 Budapest, Hungary; volk.balazs@egis.hu (B.V.); simig.gyula@egis.hu (G.S.); 5 Chemical Biology Research Group, Institute of Organic Chemistry, HUN-REN Research Centre for Natural Sciences, Magyar Tudósok Krt. 2, H-1117 Budapest, Hungary; nemeth.krisztina@ttk.hu; 6 CarboHyde Ltd., Berlini u. 47-49, H-1045 Budapest, Hungary; milo.malanga@carbohyde.com

**Keywords:** selective phosphoinositide 3-kinase delta inhibitor, chiral impurity profiling, hydroxypropyl-β-cyclodextrin chiral selector, host-guest complexation, variable temperature NMR, ^19^F NMR, ^1^H-^19^F HOESY NMR, 2D ROESY NMR, diastereomeric splitting, design of experiments

## Abstract

Idelalisib (IDE) is a marketed chiral anticancer drug administered as the *S*-enantiomer, requiring sensitive monitoring of the *R*-enantiomer to ensure enantiomeric purity. However, no dedicated capillary electrophoresis (CE) method has been reported for trace-level quantification of *R*-IDE. In this study, a cyclodextrin-mediated CE method was developed for reliable detection of the *R*-enantiomer at the 0.1% level (LOD 2 µg/mL; LOQ 5 µg/mL). Systematic screening identified hydroxypropyl-β-cyclodextrin (HP-β-CD) with an intermediate degree of substitution (DS~6.8) as the optimal chiral selector, providing efficient enantioseparation (*R_s_* up to 4.3). The method was validated according to ICH Q2(R2) guidelines, demonstrating suitable precision, accuracy, and robustness. Complementary NMR studies revealed hindered rotation of the 3-phenyl moiety and elucidated the molecular basis of enantioselectivity. Complexation with β-CD and HP-β-CD produced clear diastereomeric differentiation in both ^1^H and ^19^F NMR spectra, while the simplified ^19^F NMR profiles enabled direct enantiomer discrimination. NOESY and ROESY experiments demonstrated distinct inclusion modes, with HP-β-CD accommodating both the fluorinated aromatic ring and the 3-phenyl moiety. These interactions may account for the superior enantioseparation observed with HP-β-CD of intermediate DS. Our validated CE method addresses the distomer determination while NMR insights provide mechanistic understanding of the chiral recognition.

## 1. Introduction

**Figure 1:**
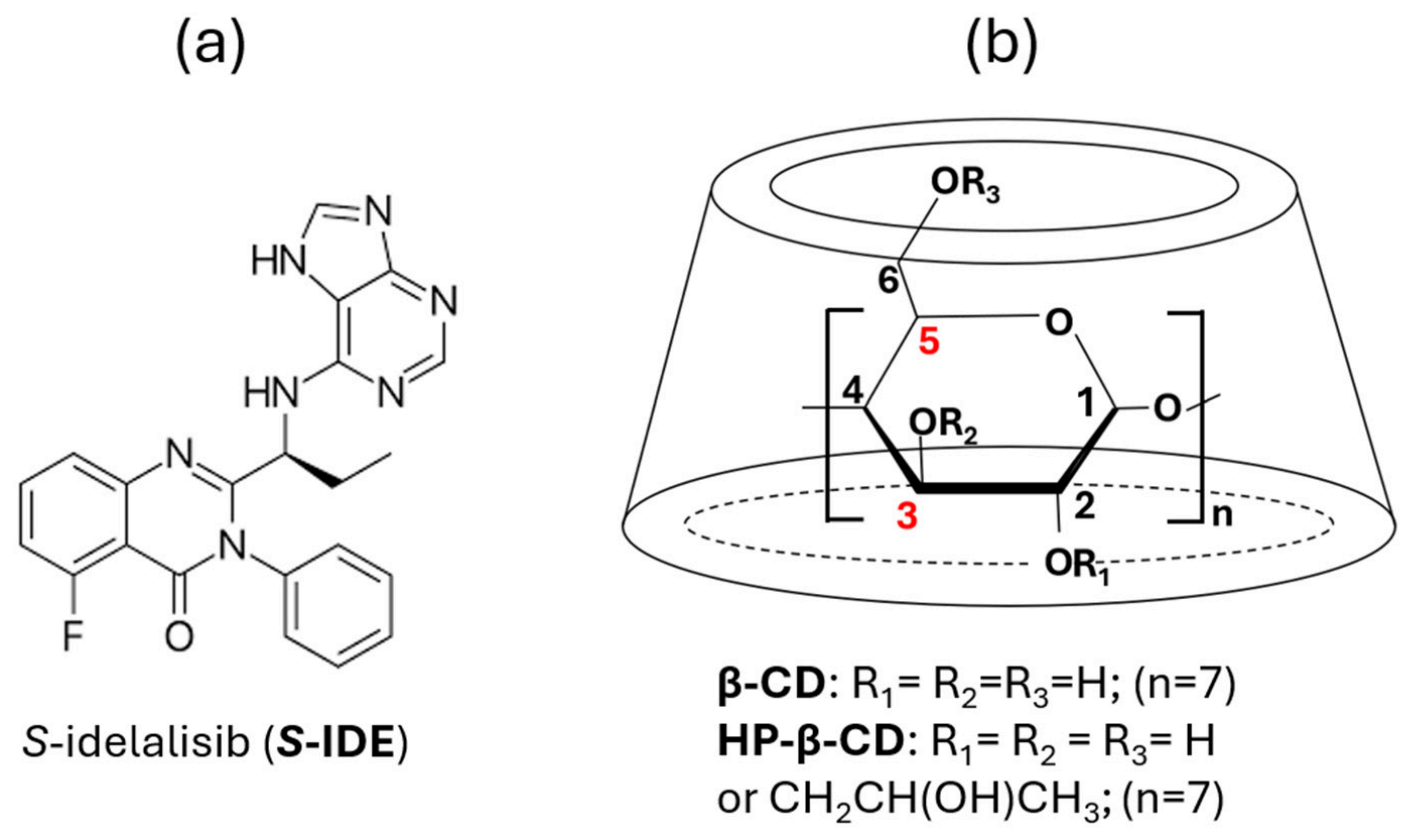
(**a**) Chemical structure of *S*-idelalisib (*S*-IDE, 5-fluoro-3-phenyl-2-[(1*S*)-1-(9*H*-purin-6-ylamino)propyl]-3*H*-quinazolin-4-one), (**b**) Schematic representation of cyclodextrins (CDs), highlighting the 3-, and 5-positions on the glucose units. Abbreviations: β-cyclodextrin (β-CD), hydroxypropyl-β-CD (HP-β-CD).

Idelalisib (IDE, Figure 1) is a selective phosphoinositide 3-kinase delta (PI3Kδ) inhibitor marketed as Zydelig^®^ for the treatment of several hematological malignancies [1,2,3]. IDE contains a single chiral centre with *S*-configuration [4], which enables selective binding to the hydrophobic hinge-region pocket of PI3Kδ [5,6,7,8,9,10]. Structural studies suggest that the *S*-enantiomer adopts the optimal binding geometry within the PI3Kδ binding pocket, whereas the *R*-enantiomer is predicted to be less favourably accommodated because of geometric constraints, potentially resulting in reduced binding affinity and selectivity [11]. Although no pharmacological data are available for the *R*-enantiomer (*R*-IDE), it is regarded as a chiral impurity from a regulatory perspective. Therefore, its level must be strictly controlled in both the active pharmaceutical ingredient and the finished product.

Several reversed-phase HPLC methods using conventional C18 columns have been described for the determination of IDE in biological matrices [12,13,14,15,16,17]. Chiral HPLC methods employing polysaccharide-based stationary phases have also been reported in the context of synthetic process development [4]. However, their application in routine quality control remains limited, as these methods were not designed or validated for the quantification of trace-level *R*-IDE. To the best of our knowledge, no dedicated analytical method, either by HPLC or capillary electrophoresis (CE), has been published for this purpose.

CE is a particularly attractive technique for chiral impurity analysis due to its high separation efficiency and low material consumption. Its flexibility allows rapid screening of chiral selectors, especially cyclodextrins (CDs, Figure 1), by simple modification of the background electrolyte (BGE) [18,19]. The enantioselective properties of CDs can be systematically tuned via chemical derivatization, enhancing transient host-guest interactions. Notably, even when both enantiomers have comparable affinity for the chiral selector, differences in the effective electrophoretic mobilities of the transient diastereomeric complexes may still enable discrimination [20]. Despite the widespread use of CDs in chiral CE, the molecular basis of enantiorecognition, including the role of inclusion geometry, analyte conformation, and the chemical modification of the CD cavity, often remains poorly understood.

In spite of its advantages, the development of chiral CE methods is inherently complex due to the simultaneous influence of multiple interdependent parameters, including buffer composition and pH, CD type and concentration, applied voltage, temperature, etc. Traditional one-factor-at-a-time optimization approaches are often insufficient to capture such interactions. In contrast, Design of Experiments (DoE) enables systematic multivariate analysis of critical factors and their combined effects, allowing efficient optimization with a reduced number of experiments.

Accordingly, the present study pursued two complementary objectives: (i) the development and ICH-compliant validation of a CD-assisted CE method for the trace-level quantification of *R*-IDE, and (ii) the elucidation of the structural basis of enantioselectivity using NMR spectroscopy.

## 2. Results and Discussion

### 2.1. Synthetic Procedures

**Scheme 1:**
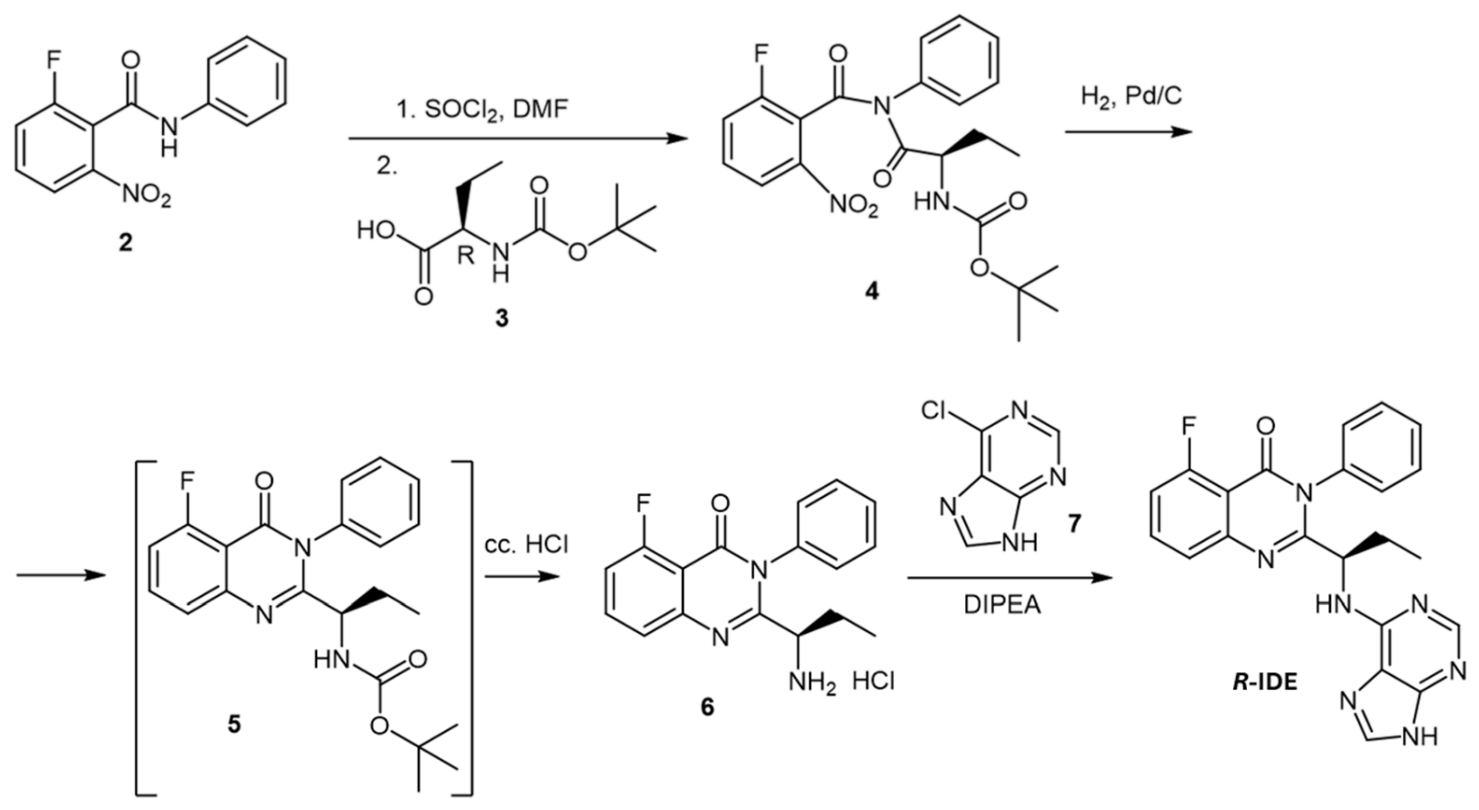
Synthesis of *R*-enantiomer of idelalisib (*R*-IDE).

Owing to its biological activity and pharmaceutical significance, the synthesis of *S*-IDE has been extensively described in the literature [4,21,22,23,24,25]. To enable CE-based enantiomeric separation studies and NMR investigations of chiral recognition, access to both enantiomers was required. However, the corresponding *R*-enantiomer (*R*-IDE) has not, to the best of our knowledge, been previously reported. Therefore, *R*-IDE was synthesized via a route analogous to that reported for *S*-IDE, employing (2*R*)-2-(*tert*-butoxycarbonylamino)butanoic acid as the chiral precursor instead of its *S*-configured counterpart (Scheme 1). The identity of *R*-IDE was confirmed by NMR and IR spectroscopy (Supplementary Material, Figures S1–S3), while its enantiomeric purity was verified by chiral CE, ensuring its suitability for subsequent analytical studies.

### 2.2. Capillary Electrophoresis

#### 2.2.1. CE Method Development

##### Initial Scouting Experiments

IDE has three reported p*K_a_* values (1.6, 3.4, and 9.8), according to the product information in the Australian Public Assessment Report issued by the Therapeutic Goods Administration (TGA), Australian Government Department of Health [26]. However, no details on their determination are provided. The heterocyclic structure of IDE and the reported p*K*_a_ values both suggest that the analyte is predominantly protonated under acidic conditions. Accordingly, two acidic background electrolytes (pH 2.5 and 3.0) were evaluated for CE separation to ensure a permanent positive charge of the analyte. At pH 2.5 (phosphate and citrate buffer), distorted peak shapes and pronounced baseline fluctuations were observed, whereas a citrate buffer at pH 3.0 provided symmetric peak shapes and good baseline stability. Organic modifiers were tested in the buffer system (methanol and acetonitrile, 10–50% *v*/*v*, without selector), but they did not improve peak shape and increased baseline noise and were therefore omitted. Consequently, pH 3.0 citrate buffer was selected for further method development.

**Figure 2:**
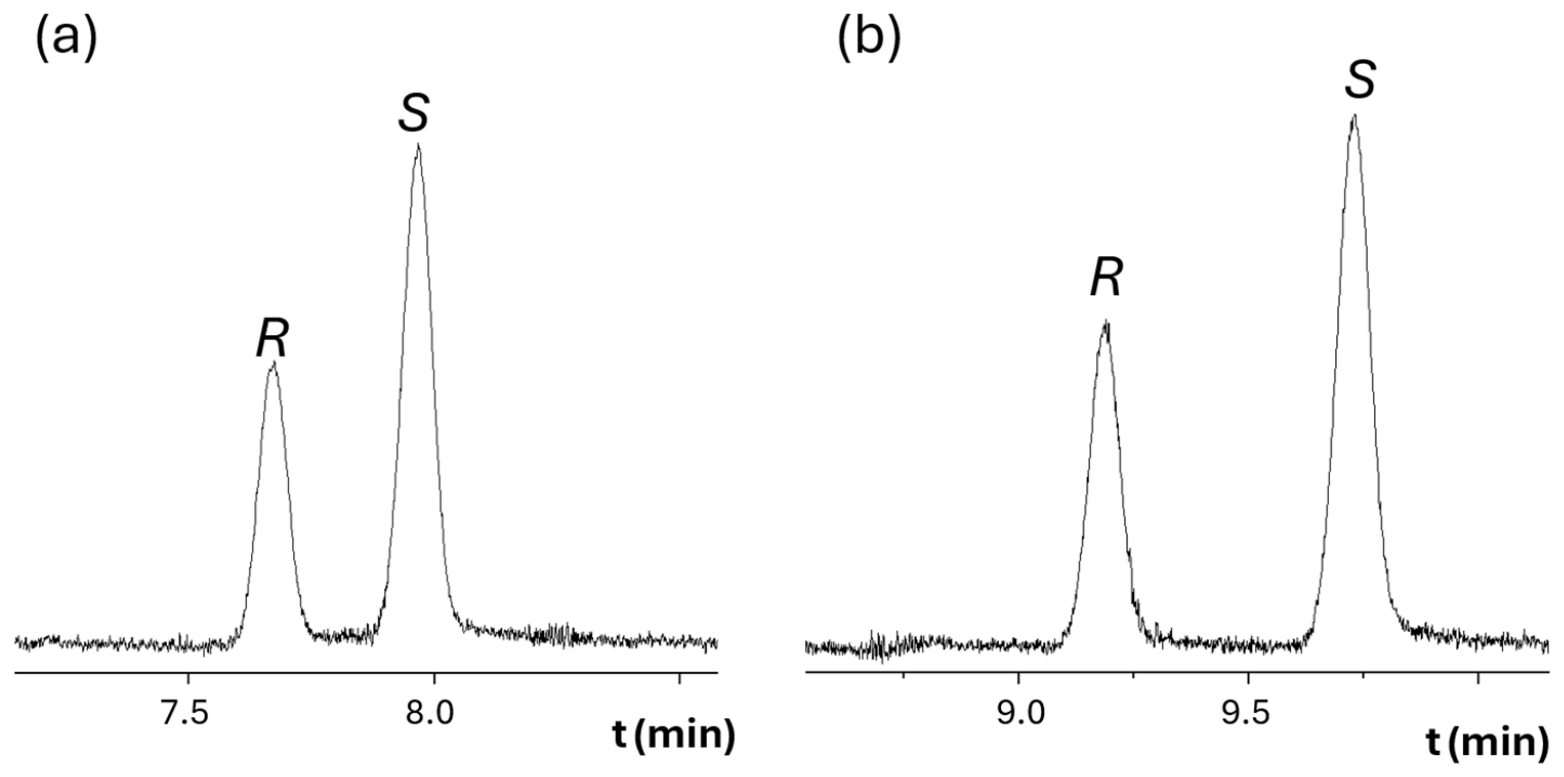
Representative electropherograms showing the enantiomer migration order in spiked samples (**a**) in the presence of 10 mM β-CD, (**b**) in the presence of 30 mM HP-β-CD. Enantiomeric ratio was set to 1:2 *R*-IDE:*S*-IDE. (20 kV, 25 °C, 214 nm).

At pH 3.0 and under standard conditions (25 °C, 20 kV), a rapid screening of CDs was conducted to identify selectors capable of achieving baseline enantiomeric separation (*R_s_* ≥ 1.5). Native β-CD (10 mM) provided *R_s_* 2.5 with the desired enantiomer migration order (EMO), in which the distomer migrated first, indicating that the cavity size of β-CD is well suited for the enantiorecognition of IDE (Figure 2).

**Figure 3:**
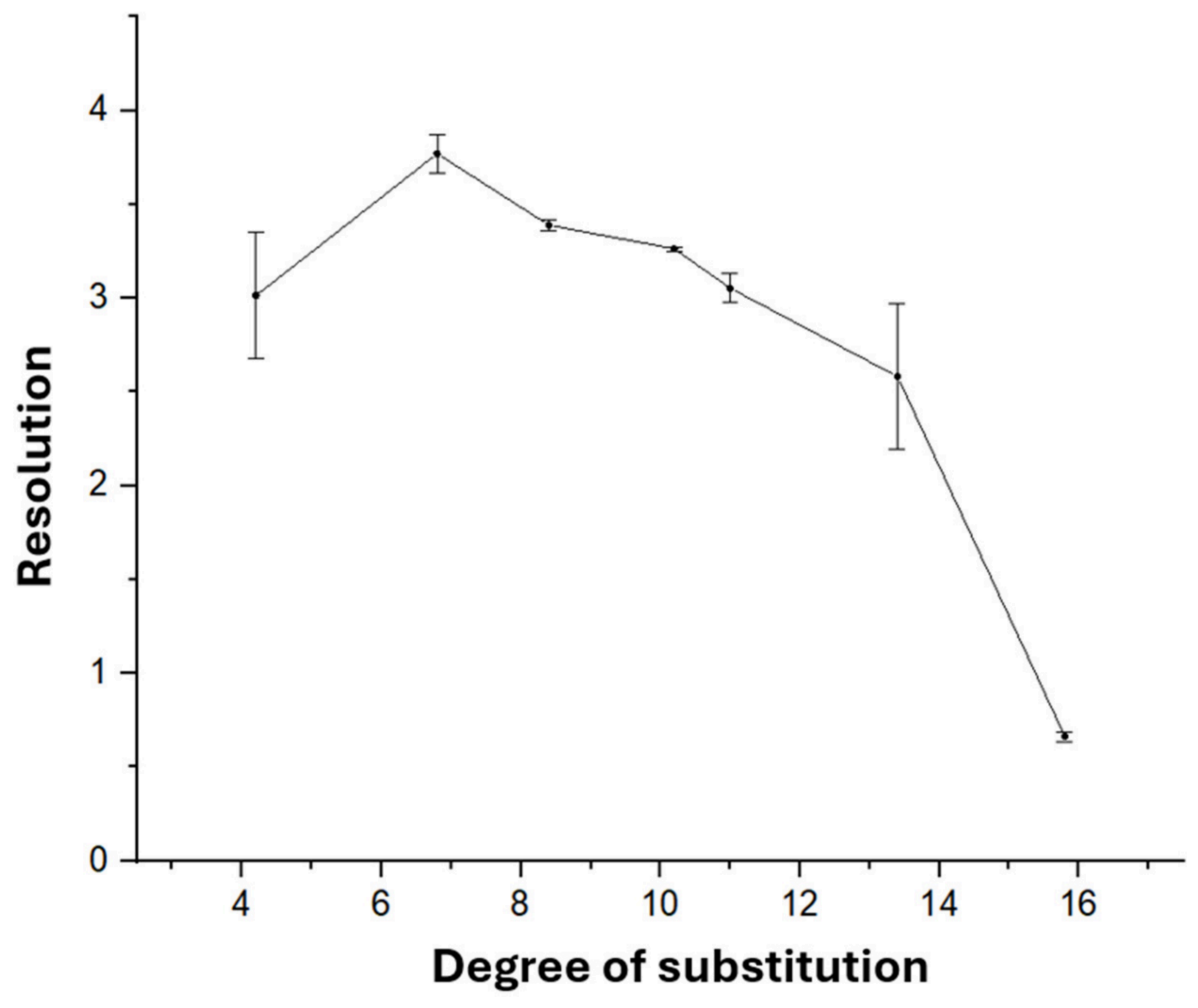
Representative plot of enantiomeric resolution versus the degree of substitution of HP-β-CD for IDE under fixed separation conditions: 10 mM citrate buffer (pH 3.0), 30 mM HP-β-CD, 25 kV, 25 °C, 214 nm.

Although native β-CD already provided baseline separation, further improvement in resolution was pursued to ensure adequate separation at 0.1% impurity level by investigating derivatized CDs. Hydroxypropyl-β-cyclodextrin (HP-β-CD) was studied, as its enhanced aqueous solubility (45–50% *w*/*v*) allows the use of higher selector concentrations. HP-β-CDs are randomly substituted at the O2, O3, and O6 positions of the glucose units, resulting in a heterogeneous mixture characterized by different degrees of substitution (DS) as well as various patterns of substitution [27]. Theoretical studies suggest that increasing DS enhances conformational flexibility and reduces structural symmetry, while the effective cavity size depends on the substitution pattern: O2- and O6-substitution generally widen the cavity, whereas O3-substitution may partially narrow the rim due to inward-oriented groups [28]. Based on these considerations, HP-β-CDs with DS values ranging from 4.2 to 15.8 were systematically screened to identify the optimal DS for achieving maximum enantiomeric resolution. Among these, HP-β-CD with DS 6.8 provided the highest resolution (*R_s_* 3.7), suggesting that an intermediate DS provides the most favourable balance between cavity accessibility and conformational flexibility (Figure 3). Consequently, this selector was identified as optimal during the scouting experiments and was therefore fixed for the subsequent DoE optimization.

In addition to selector screening, preliminary experiments were performed to define the relevant factor ranges for the subsequent DoE study. Based on the analyte ionization behaviour and the superior peak shape, the buffer pH was fixed at 3.0. Separation temperature was also fixed at 25 °C, as lower temperatures resulted in broader peaks, whereas higher temperatures promoted excessive Joule heating, which was considered unfavourable for capillary performance. Because the low-pH system exhibited relatively slow migration, the applied voltage was investigated within a higher range to maintain acceptable analysis times. The concentration ranges of HP-β-CD and buffer were selected to ensure at least baseline separation. Excessively high buffer concentrations were avoided because they caused pronounced baseline noise and fluctuations.

##### Method Optimization Using DoE

Based on the scouting results, HP-β-CD (DS~6.8), buffer pH (3.0, citrate) and capillary temperature (25 °C) were fixed, and the remaining three factors (applied voltage, HP-β-CD concentration, and buffer concentration) were selected for multivariate optimization using a Box–Behnken design (Table S1). Each factor was studied at three levels: voltage at 20, 25, and 30 kV; HP-β-CD concentration at 10, 20, and 30 mM; and buffer concentration at 10, 25, and 50 mM. The design comprised 15 runs including three centre-point replicates for estimation of pure error (Table S2). Enantiomeric resolution (*R_s_*) and distomer peak symmetry were recorded as response variables.

Buffer concentration was the most influential factor among those tested, with significant linear (*p* = 0.005) and quadratic (*p* = 0.007) contributions, indicating a pronounced non-linear dependence of resolution on ionic strength. Among the factor interactions, the quadratic voltage × linear buffer concentration interaction (*p* = 0.012) reached significance. For peak symmetry, the linear term of buffer concentration was again the most significant (*p* = 0.011), followed by the linear term of CD concentration (*p* = 0.046). Notably, the CD concentration × buffer concentration interaction was significant for symmetry (*p* = 0.040) though not for resolution (Tables S3 and S4).

**Figure 4:**
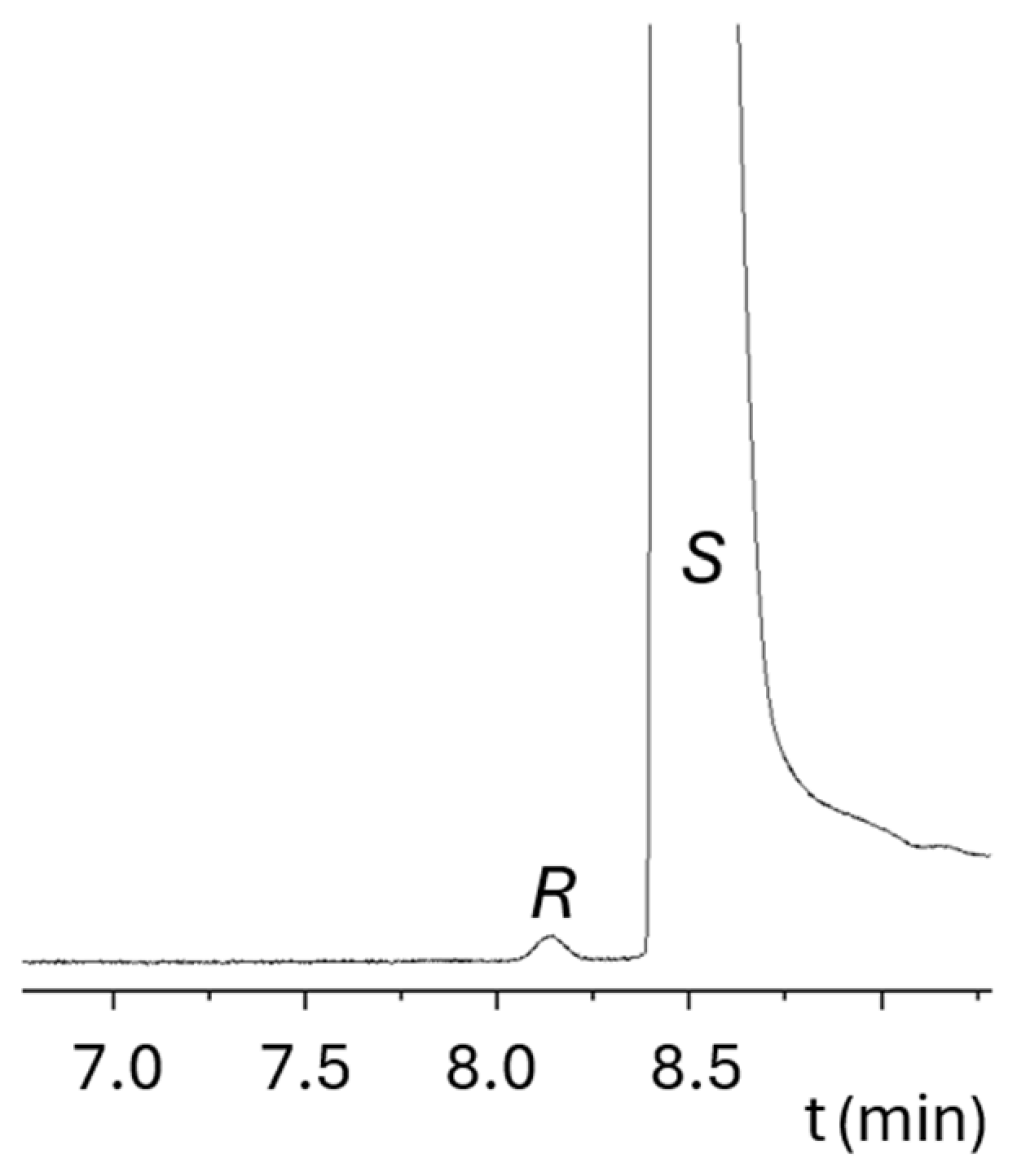
Representative electropherogram illustrating the detection of 0.1% (5 µg/mL) *R*-IDE impurity in the presence of 5000 µg/mL *S*-IDE. Separation conditions: 20 mM HP-β-CD (DS~6.8) in 25 mM citrate buffer (pH 3.0), 25 kV, 25 °C, 214 nm.

The centre-point conditions were selected based on a hierarchical evaluation in which enantiomeric resolution was first maximized, followed by the choice of the condition exhibiting superior peak symmetry among those with comparable *R_s_* values. The centre-point settings (20 mM HP-β-CD, DS~6.8; 25 mM citrate buffer, pH 3.0; 25 kV applied voltage; 25 °C capillary temperature) offered the best combination of enantiomeric resolution and distomer peak symmetry (max. *R_s_* 4.36), enabling the reliable determination of 0.1% (5 μg/mL) *R*-IDE impurity in the presence of 5000 μg/mL *S*-IDE (Figure 4). The corresponding optimization results are provided in Supplementary Materials Table S2 and Figures S4 and S5.

##### Robustness

To assess method robustness, a Plackett–Burman design was employed in which five analytical parameters were deliberately varied within their normal operating ranges: HP-β-CD concentration (20 ± 1 mM), buffer concentration (25 ± 1 mM), voltage (25 ± 1 kV), pH (3.0 ± 0.1), and temperature (25 ± 1 °C) (Table S5). The design comprised eight factorial runs and three centre-point replicates (Table S6). Resolution was monitored as response. Pareto analysis of standardized effects (Figure S6) confirmed that none of the five factors had a statistically significant influence on the resolution at the 95% confidence level. Furthermore, the enantiomer migration order has not changed, confirming that the method is robust.

#### 2.2.2. CE Method Validation

The optimized CE method was validated in accordance with the International Council for Harmonisation (ICH) Q2(R2) guideline [29,30] for the determination of *R*-IDE as an enantiomeric impurity in the presence of a large excess of *S*-IDE (5.0 mg/mL). Method sensitivity was first assessed to verify its capability for low-level impurity monitoring. The limits of detection (LOD) and quantification (LOQ) were established at 2 µg/mL and 5 µg/mL, respectively, based on signal-to-noise ratios of 3 and 10. These values were confirmed to be adequate for controlling the 0.1% impurity level (Figure 4, Table 1).

Considering the LOQ of the method, the linearity range was established between 5 and 25 µg/mL for *R*-IDE, using five concentration levels. This range corresponds to impurity levels of 0.1–0.5% relative to the concentration of the *S*-IDE active pharmaceutical ingredient (API). The calibration curve for *R*-IDE demonstrated excellent linearity, see Table 1 and Supplementary Material Table S7 and Figure S7.

Method accuracy and precision for *R*-IDE quantification were evaluated at three concentration levels covering the validated range. Mean recovery values (*n* = 3 for each level) ranged between 100.3% and 102.0%, with relative standard deviations decreasing from 6.4% at the LOQ level to 2.7% at the upper range. Intraday repeatability and intermediate precision on two consecutive days were below 4% and 6% RSD, respectively. Migration time repeatability was assessed on three different days, with all RSD values below 4% and intermediate precision not exceeding 7% (Table 1 and Supplementary Material Tables S8–S11).

Overall, these validation results demonstrate that the developed CE method provides sufficient sensitivity, excellent linearity, and reliable accuracy and precision for the quantitative determination of *R*-IDE in a quantity of 0.1–0.5% in the presence of API excess. While the validated CE method may provide a robust analytical tool for enantiomeric impurity control, the molecular basis of the observed enantioselectivity remained unclear. Therefore, complementary NMR investigations were undertaken to elucidate the host–guest interactions responsible for chiral recognition and to explore whether NMR could provide an alternative means of enantiomer discrimination.

### 2.3. NMR Studies

The conformational behaviour of IDE and its interactions with CDs were investigated by NMR spectroscopy to gain insight into the dynamic behaviour of the molecule and the structural basis of the host-guest complex formations.

#### 2.3.1. Variable Temperature NMR Study of IDE

The complete NMR signal assignment of IDE was previously reported in DMSO-*d*_6_ at 353 K [4], although the rationale for using an elevated temperature was not discussed. To obtain detailed structural information under ambient conditions, a set of 2D NMR experiments (COSY, HSQC, HMBC, NOESY; Figures S8–S17) was recorded in DMSO-*d*_6_ at 298 K.

**Figure 5:**
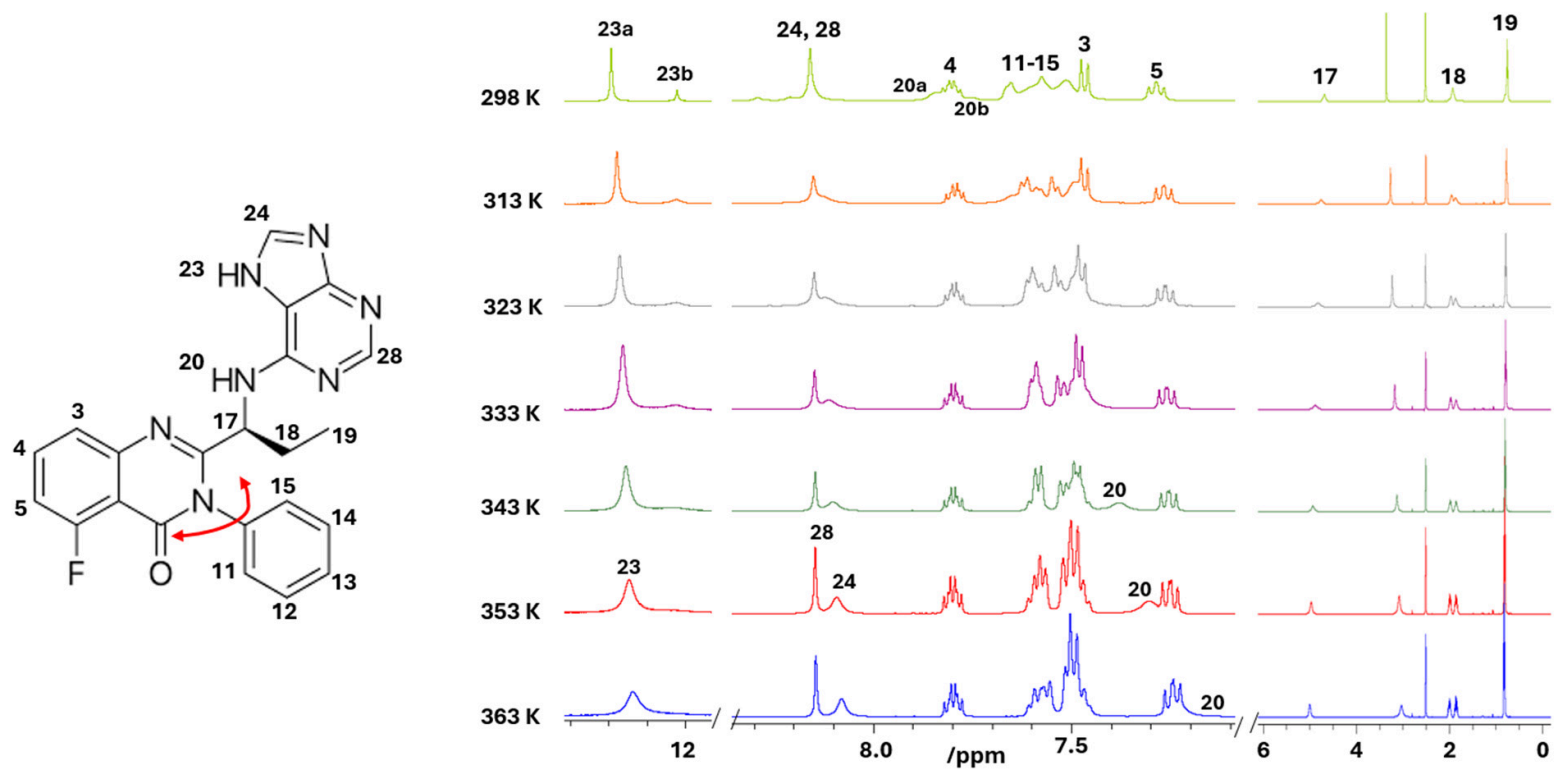
Molecular structure of *S*-IDE, with atom numbering, highlighting the restricted rotation around the *C*-*N* bond (**left**). Representative ^1^H NMR spectra of *S*-IDE recorded at 500 MHz in DMSO-*d*_6_ over the temperature range of 298–363 K (**right**).

At 298 K, broad resonances were observed in the ^1^H NMR spectrum for the aromatic protons of the 3-phenyl moiety (H11–H15), the aliphatic side chain (notably H17 and H18), the aromatic protons of the heterocycle (H24 and H28), and the NH protons (Figure 5, Supplementary Material, Table S12, Figures S8–S17), consistent with slowly interconverting atropisomeric species in solution [31]. In contrast, the aromatic signals of the fluorinated ring remained predominantly sharp. In addition, the purine NH (H23) displayed two sets of resonances in a 1:6.4 ratio (Figure 5 and Figure S17), suggesting the presence of two slowly interconverting species, which may arise from purine tautomerism or conformational exchange.

Upon increasing the temperature, a significant signal sharpening was observed (Figure 5), indicating faster exchange on the NMR timescale. However, the H24 signal remained broad. Under these conditions, the diastereotopic nonequivalence of the H18 protons became more clearly resolved, while the H23 signal coalesced into a broad singlet. The broadening of the aromatic signals (H11–H15) is attributed to hindered rotation about the exocyclic *C*-*N* bond linking the quinazolinone nitrogen to the phenyl ring (Figure 5). Although hindered *C*-*N* rotation is well documented [32,33,34,35], such dynamic behaviour has not previously been reported for IDE.

The possibility of hindered amide rotation involving the quinazolinone C4=O group cannot be excluded. However, interpretation of the ^13^C NMR data is complicated by ^19^F-^13^C coupling, which obscures the identification of any additional dynamic contributions (Figure S14 and Table S12).

In aqueous solution (D_2_O, pD 3.0), the 3-phenyl proton signals were also broad and the ortho-proton nonequivalence at 298 K again indicated restricted rotation (Supplementary Material Figures S18 and S19). The diastereotopic H18 protons were more clearly resolved at 298 K than in DMSO-*d*_6_. The purine HN proton (H23) was not detected, consistent with rapid H/D exchange in D_2_O. Temperature-dependent sharpening further confirmed faster exchange between the species in equilibrium. These observations are consistent with a slow exchange on the NMR timescale.

To obtain quantitative kinetic data, variable-temperature ^1^H NMR spectra were initially recorded in D_2_O over the temperature range of 5–50 °C using a 700 MHz spectrometer. The high-field instrument was selected to maximize spectral resolution and provide an adequate signal-to-noise ratio for the poorly water-soluble IDE. However, these spectra proved unsuitable for line-shape analysis. Therefore, DMSO-*d*_6_ was selected as an alternative solvent, as it exhibited the same dynamic process while providing spectra suitable for quantitative analysis. Variable-temperature experiments were subsequently performed on a 500 MHz spectrometer over the temperature range of 298–363 K.

**Figure 6:**
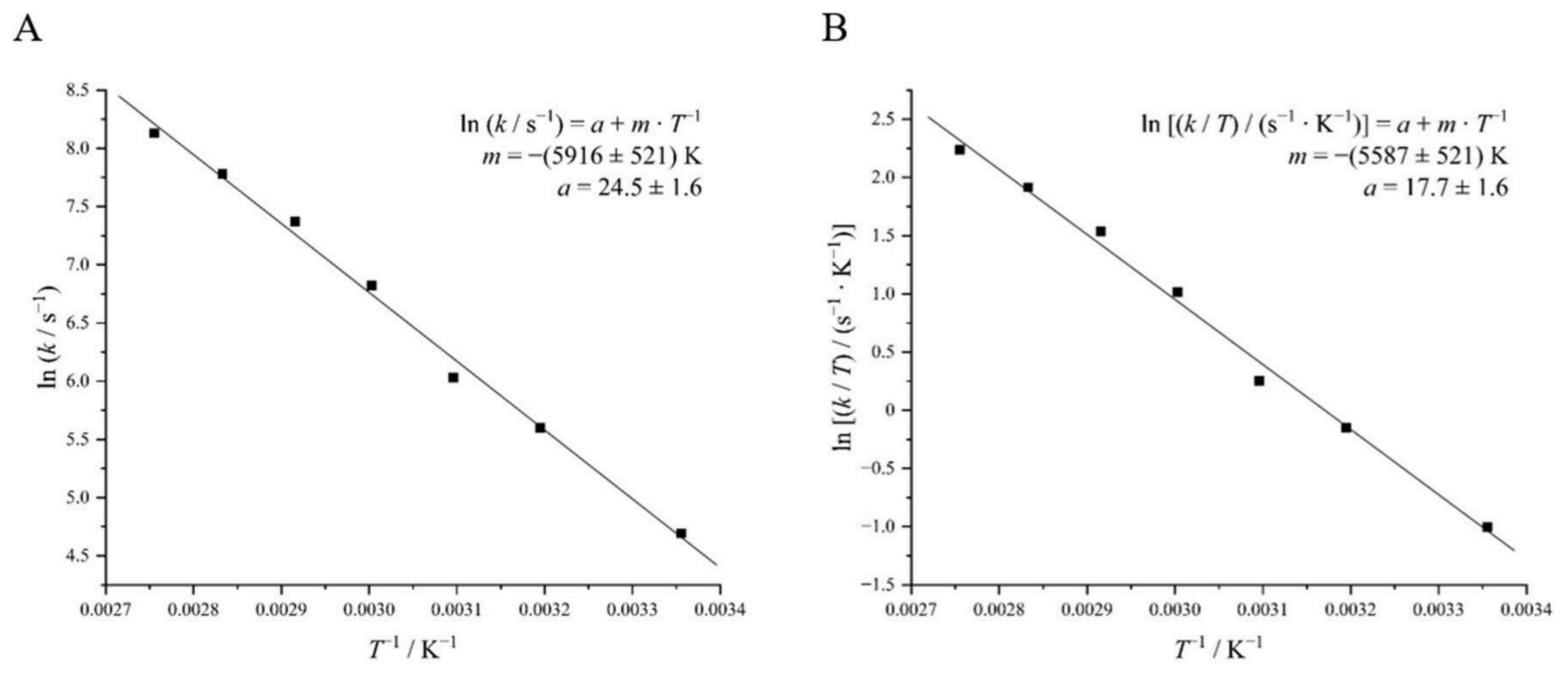
(**A**) Linearized Arrhenius plot for the determination of the activation energy. The solid line represents the least-squares linear fit. Error estimates correspond to 95% confidence intervals, and the coefficient of determination was *r*^2^ = 0.99417. (**B**) Linearized Eyring plot for the determination of the activation enthalpy and activation entropy. The solid line represents the least-squares linear fit. Error estimates correspond to 95% confidence intervals, and the coefficient of determination was *r*^2^ = 0.99345.

Because the aromatic ^1^H resonances exhibited substantial overlap, complete line-shape simulations were not feasible. Instead, the well-resolved aliphatic side-chain resonances H18a, H18b, and H19 were selected for analysis and modelled as an A_3_MNP spin system (Figures S20–S26). The resulting temperature-dependent rate constants were used to construct Arrhenius and Eyring plots, which were analyzed by linear regression (Figure 6).

Using the equations described in Section 3.2.3, the activation parameters were determined. The activation energy ($E_{a}$) obtained from the Arrhenius plot was (48.1 ± 4.3) kJ mol^−1^. Analysis of the Eyring plot yielded an activation enthalpy ($∆H^{‡}$) of (46.5 ± 4.3) kJ mol^−1^ and an activation entropy ($∆S^{‡}$) of (−50 ± 13) J mol^−1^ K^−1^. The corresponding Gibbs free energy of activation ($∆G^{‡}$) calculated at 298.15 K, was (61.4 ± 5.8) kJ mol^−1^.

#### 2.3.2. NMR Study of IDE-CD Complexes

Following complete ^1^H signal assignment of IDE in D_2_O at pD 3.0, its complexation with CDs was investigated under conditions comparable to those used in CE separation (Supplementary Material, Table S13 and Figures S27–S34). To enable unambiguous signal assignment and minimize spectral crowding, the individual enantiomers of IDE were examined in the presence of β-CD and HP-β-CD prior to analysis of racemic mixtures.

**Figure 7:**
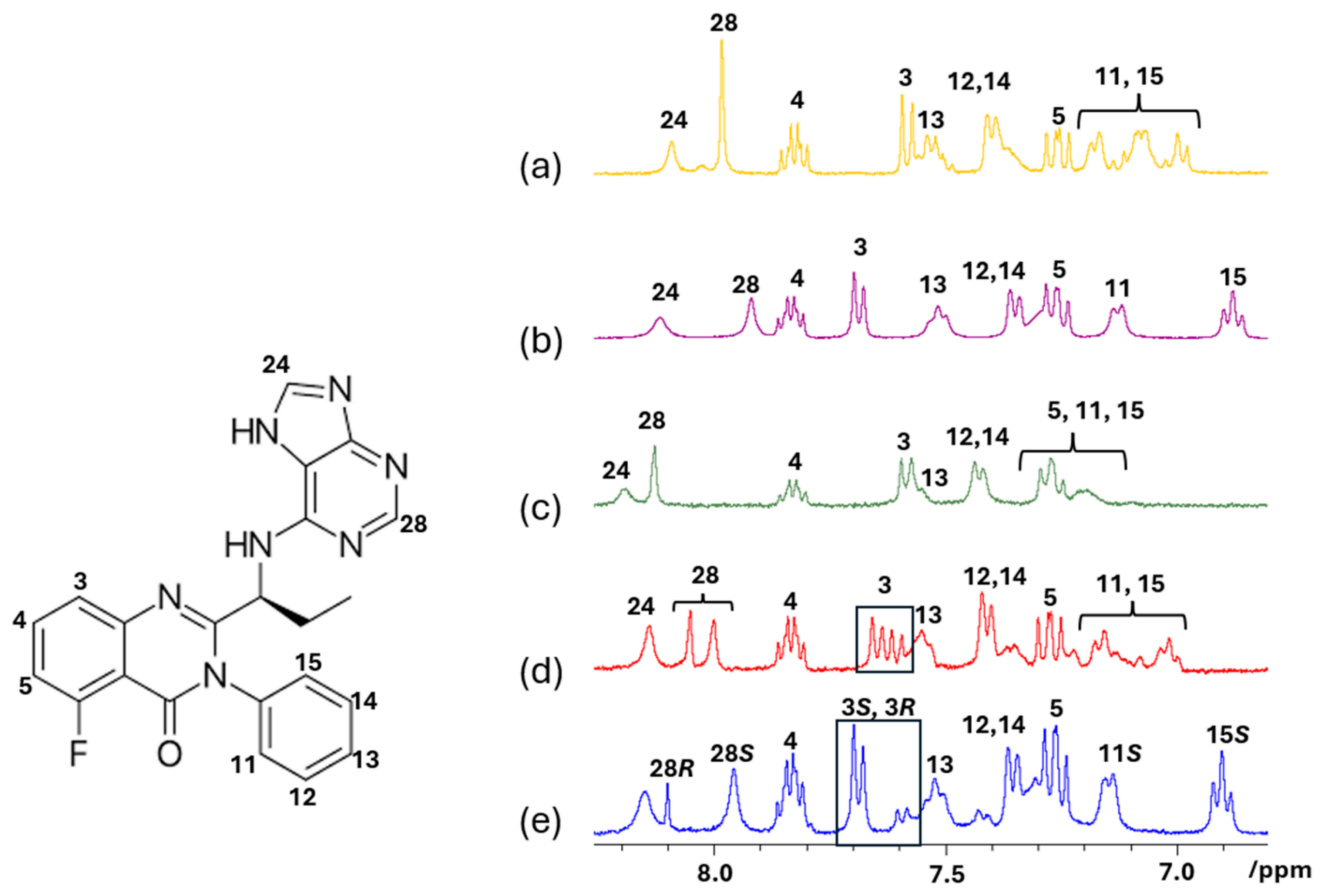
Chemical structure of *S*-IDE, with atom numbering (**left**). Representative ^1^H NMR spectra of (**a**) *S*-IDE in buffer, (**b**) *S*-IDE + HP-β-CD (1:10), (**c**) *R*-IDE + HP-β-CD (1:10), (**d**) racemic IDE + HP-β-CD (1:10), and (**e**) *R*-IDE + HP-β-CD (1:10) spiked with *S*-enantiomer (4:1 *S*:*R* ratio). Spectra were recorded in D_2_O (pD 3.0) at 298 K on a 400 MHz spectrometer.

Upon complexation with both β-CD and HP-β-CD, a pronounced sharpening of the 3-phenyl aromatic signals, particularly H11 and H15, was observed for *S*-IDE (Figure 7 and Supplementary Material Figures S35 and S36). This phenomenon is consistent with preferential stabilization of a specific rotameric state upon host-guest binding. In contrast, the corresponding resonances of *R*-IDE remained broadened (Figure 7 and Supplementary Material Figures S35 and S36), similar to those observed in buffer solution, suggesting persistence of conformational exchange.

Subsequent analysis of racemic and spiked mixtures revealed clear enantiomeric differentiation at the H3 resonance (Figure 7 and Supplementary Material Figures S35 and S36). Assignment using a non-equimolar *S*:*R* mixture (4:1) showed systematic downfield shifts for *S*-IDE and upfield shifts for *R*-IDE upon CD binding, indicating distinct chemical environments for the corresponding diastereomeric complexes (Figure 7 and Supplementary Material Figures S35 and S36).

As IDE contains a fluorine atom on the aromatic quinazolinone ring, ^19^F NMR spectroscopy [36] was employed to probe CD complexation in a site-specific manner. Changes in the fluorine chemical shift upon binding provide direct information on interactions occurring near the fluorinated ring.

**Figure 8:**
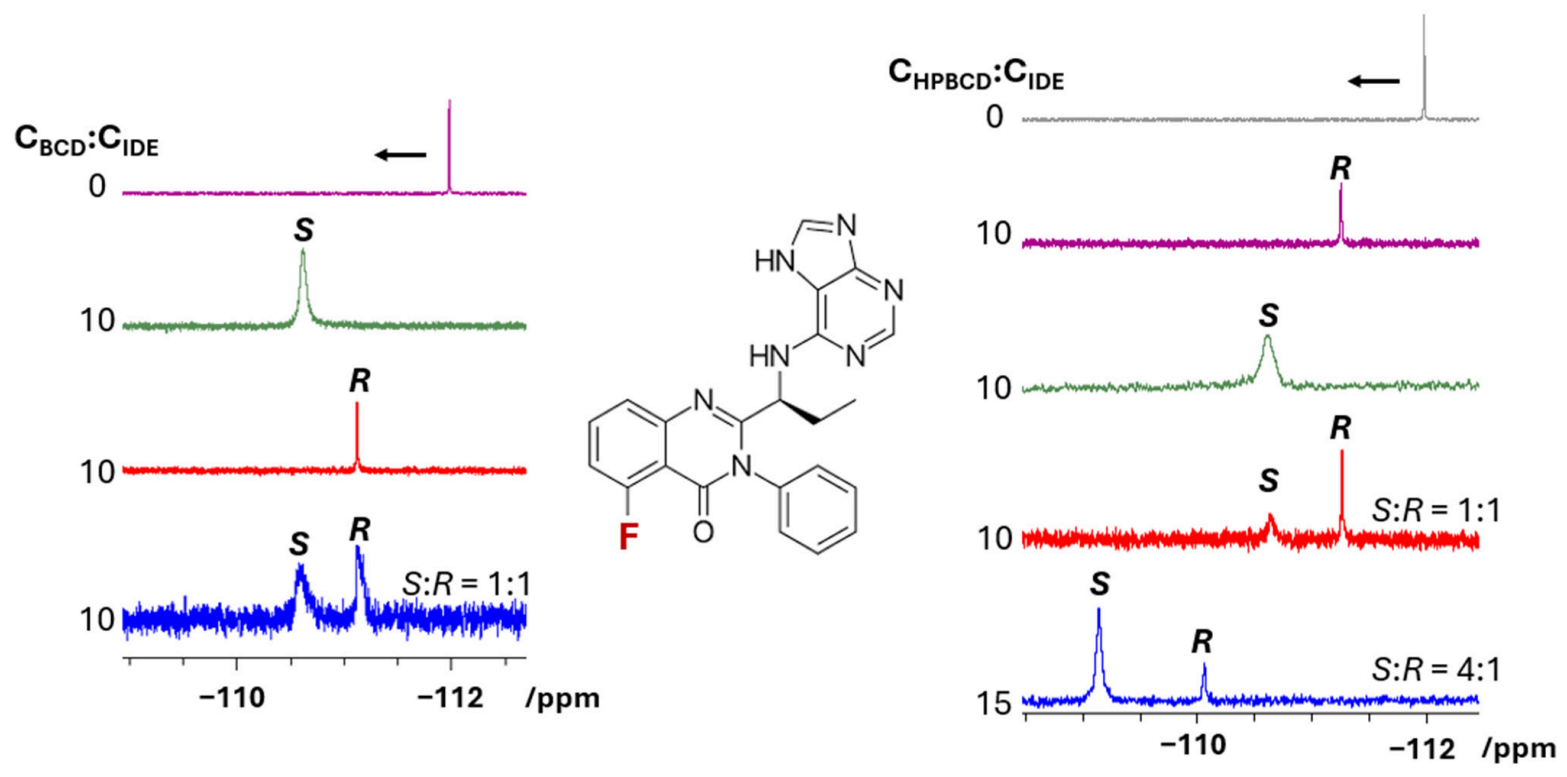
Representative ^19^F NMR spectra of IDE and its CD complexes (D_2_O; pD 3.0; 376 MHz; 298 K). (**Left**): β-CD systems. (**Right**): HP-β-CD systems. Molecular structure of *S*-IDE, with the fluorine atom highlighted, is shown in the centre. Additional experimental conditions are described in Section 3.2.3.

In the presence of β-CD (1:10 analyte:CD ratio), the *R*-enantiomer exhibited a 0.9 ppm downfield shift, while the *S*-enantiomer shifted by 1.4 ppm, resulting in a 0.5 ppm separation between the fluorine signals (Figure 8). With HP-β-CD (1:10 ratio), the *R*-enantiomer shifted by 0.7 ppm and the *S*-enantiomer by 1.4 ppm, yielding a 0.6 ppm separation. Increasing the HP-β-CD concentration to a 1:15 ratio further enhanced the effect: the *R*-enantiomer shifted by 2.0 ppm and the *S*-enantiomer by 2.8 ppm, producing a 0.8 ppm separation (Figure 8).

In both β-CD and HP-β-CD systems, the *S*-enantiomer exhibited a broader fluorine resonance, while the *R*-enantiomer displayed a sharper signal, indicating distinct exchange regimes (slow vs. fast). These findings provide direct evidence for enantioselective host-guest interactions involving the fluorinated aromatic ring. Comparing the ^1^H and the ^19^F NMR spectra, the latter showed a markedly reduced spectral crowding, highlighting its efficacy as a sensitive probe of enantiomer discrimination. Importantly, the well-resolved and simplified ^19^F NMR profiles obtained in the presence of CDs suggest that this approach may provide a convenient platform for rapid enantiomeric excess (ee) determination without the need for separation technique. Although quantitative ee analysis by ^19^F NMR was beyond the scope of the present study, the observed signal separation indicates promising potential for future applications.

ROESY, NOESY, and HOESY experiments were performed to define the inclusion mode. Cross-peaks between IDE resonances and the inner-cavity protons (H3, H5) of the CDs confirm spatial proximity, supporting a host-guest complex formation.

At 298 K (pD 3.0), the β-CD H5 and H6 resonances overlapped (Supplementary Material Figure S37). Nevertheless, the β-CD H5 signal was unambiguously assigned to 3.79 ppm using the ROESY spectrum (Figure S38). Upon heating to 313 K, the H5 and H6 resonances became resolved, and the H5 signal shifted to 3.94 ppm (Figures S39 and S40). ROESY cross-peaks were then observed between β-CD H5 and the IDE H3–H5 protons, as well as between β-CD H3 and IDE H3 (Figures S37 and S39). These correlations indicate that the fluorinated aromatic ring of the guest molecule is positioned in close proximity to the inner cavity protons of β-CD.

**Figure 9:**
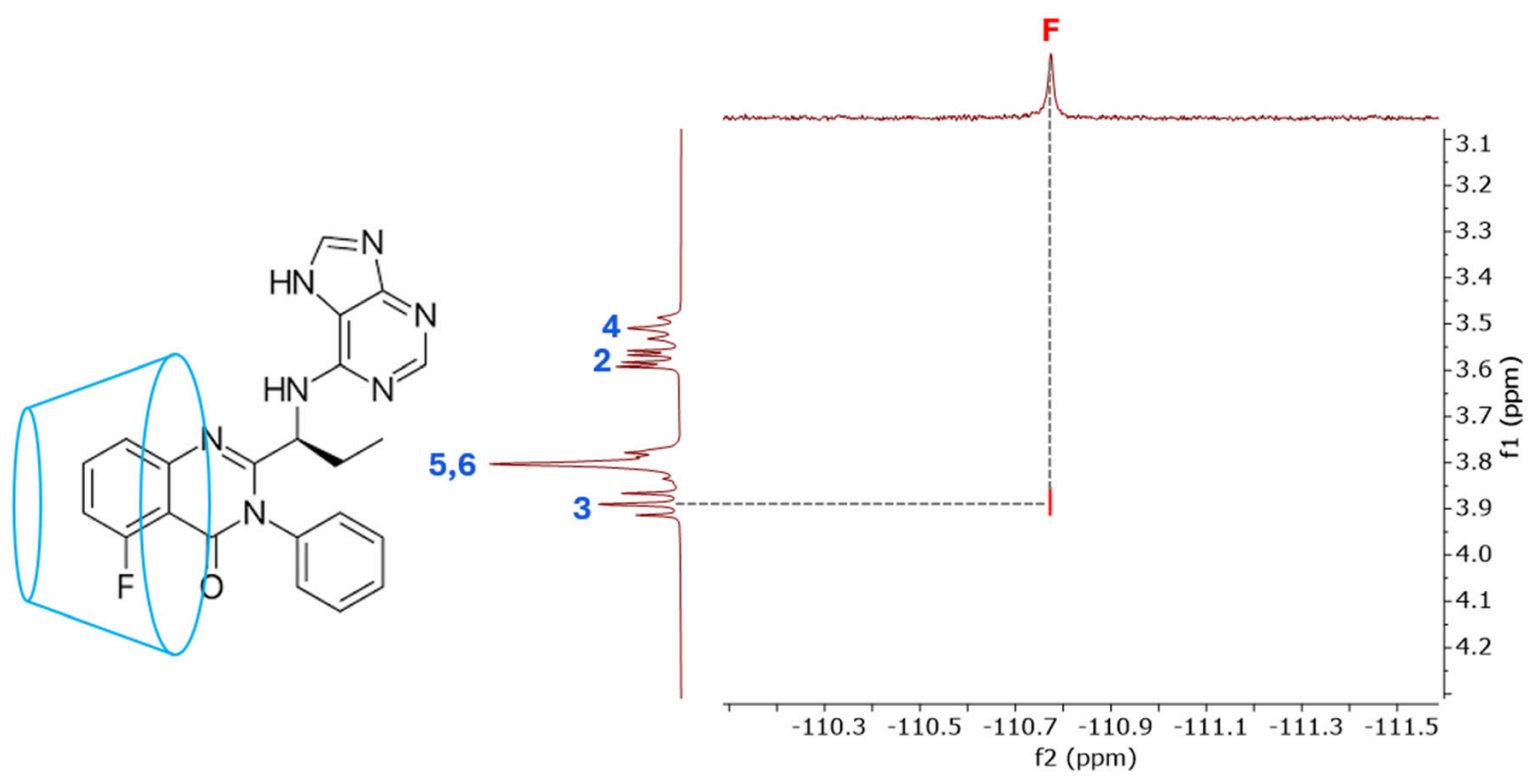
Molecular structure of *S*-IDE, highlighting the fluorinated quinazolinone moiety involved in β-CD interaction (**left**). Partial HOESY spectra of IDE and β-CD (**right**). Red: IDE fluorine resonance; Blue: β-CD resonances. (1:10; pD 3.0; 400 MHz; 298 K).

HOESY experiments further revealed an extremely weak ^19^F-H3 cross-peak above the noise level (Figure 9), providing additional evidence for spatial proximity of the fluorinated aromatic ring to the β-CD cavity. As the H3 protons are located near the wider secondary rim of β-CD, these combined ROESY and HOESY findings indicate preferential inclusion of the fluorinated aromatic ring from the secondary side (Figure 9).

**Figure 10:**
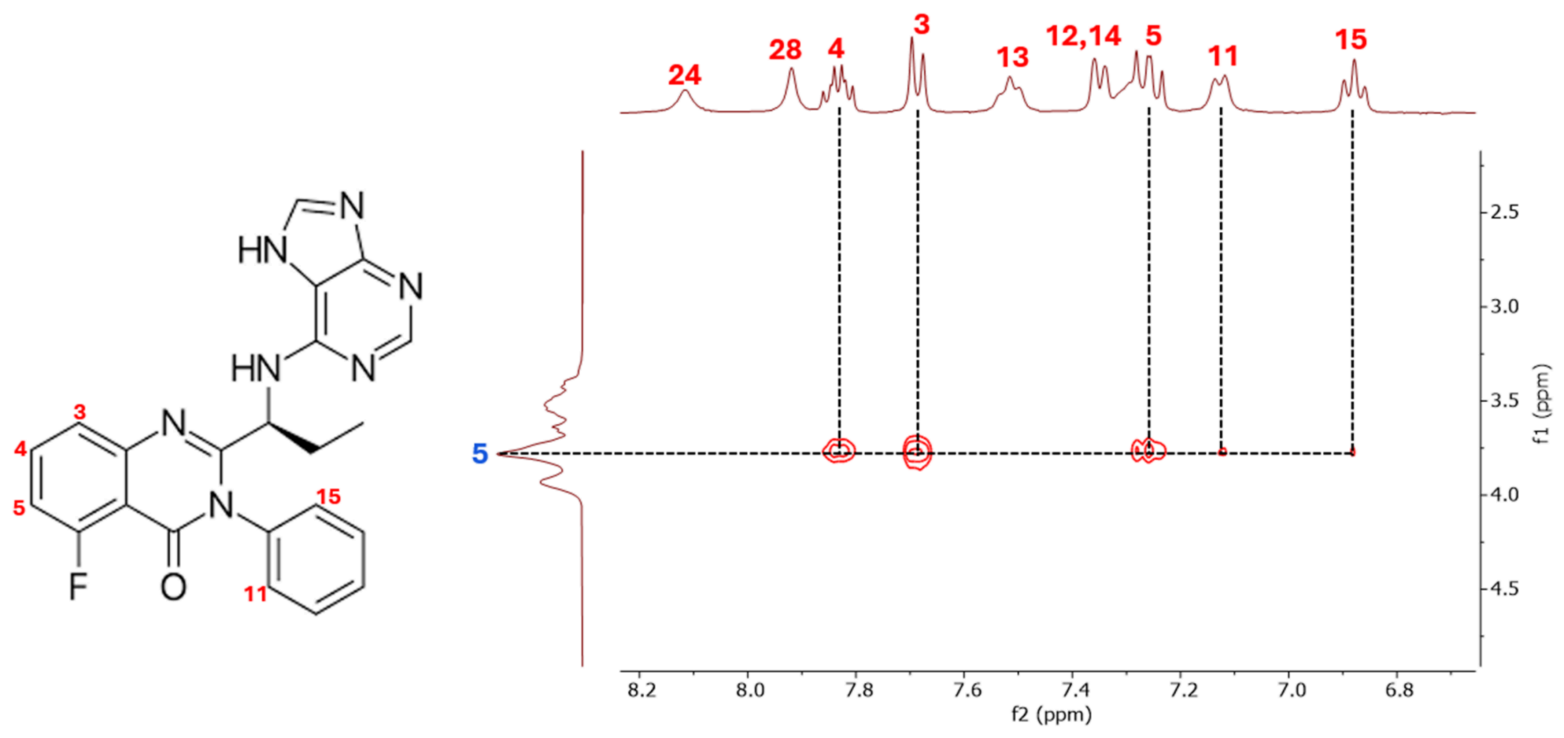
Molecular structure of *S*-IDE, highlighting the fluorinated quinazolinone moiety and the 3-phenyl ring involved in HP-β-CD interaction (**left**). Partial NOESY spectra of *S*-IDE and HP-β-CD (**right**). Red: IDE resonances; Blue: HP-β-CD resonances. (1:10; pD 3.0; 400 MHz; 298 K).

In HP-β-CD complexes, NOESY cross-peaks were observed for both the fluorinated aromatic ring and the 3-phenyl protons (H11, H15), indicating spatial proximity of both aromatic moieties to the CD cavity (Figure 10). Cross-peaks were detected exclusively with the H5 proton of HP-β-CD, whose assignment was confirmed by multiplicity-edited HSQC (Supplementary Material Figure S41). These data suggest preferential interaction of the aromatic moieties toward the primary rim (Figure 10).

Because both rings are capable of interacting with the HP-β-CD cavity, these may indicate 1:2 or 2:1 host-guest stoichiometry. A Job’s plot was therefore recorded to examine the overall complexation behaviour. Although the maximum occurred at a molar fraction of approximately 0.5 and 0.67 (Figure S42), the chemical-shift changes were small (∆δ of only a few ppb), limiting the reliability of assigning a unique stoichiometry. Therefore, rather than indicating an exclusive 2:1 IDE:HP-β-CD complex, the data are more consistent with the coexistence of 1:1 and 1:2 host-guest species in solution.

Overall, the results reveal distinct structural preferences. In β-CD complexes, the fluorinated aromatic ring is predominantly included within the cavity through the secondary rim, oriented toward the wider opening. In contrast, HP-β-CD complexes show different binding modes. The interaction can involve the fluorinated aromatic ring, the 3-phenyl ring, or both, with inclusion occurring through the primary rim (the narrower opening). These distinct inclusion modes provide a molecular-level explanation for the enhanced enantioselectivity observed with HP-β-CD in CE.

## 3. Materials and Methods

### 3.1. Materials

Native β-CD, randomly substituted HP-β-CDs with different DS (DS~4.2, 6.8, 8.4, 10.2, 11.0, 13.4 and 15.8) were supplied by CarboHyde Ltd. (Budapest, Hungary) and CycloLab Ltd. (Budapest, Hungary). Additional reagents used for buffer preparation, rinsing, or as sample solvents, including citric acid, orthophosphoric acid, sodium hydroxide, methanol, and acetonitrile, were of analytical grade and purchased from a commercial supplier (Sigma-Aldrich, Budapest, Hungary). Bidistilled Millipore water (Burlington, MA, USA) was used throughout all experiments.

### 3.2. Methods

#### 3.2.1. Synthetic Procedures

Synthesis of *tert*-butyl [(1*R*)-1-{[(2-fluoro-6-nitrophenyl)carbonyl](phenyl)carbamoyl}propyl]carbamate (**4**)

A mixture of 2-fluoro-6-nitro-*N*-phenylbenzamide (**2**, 9.10 g, 35.0 mmol), SOCl_2_ (19.0 mL, 262.5 mmol) and DMF (2 drops) was stirred at 80 °C for 6 h. It was evaporated in vacuo. The residue was dissolved in CH_2_Cl_2_ (35 mL) and it was slowly added to a solution of (2*R*)-2-(*tert*-butoxycarbonylamino)butanoic acid (**3**, 9.25 g, 45.5 mmol) and TEA (6.31 mL, 45.5 mmol) in CH_2_Cl_2_ (45 mL) at 0 °C and it was stirred at rt overnight. It was washed with water (50 mL), aqueous NaHCO_3_ solution (5 w%, 50 mL) and aqueous citric acid solution (5 w%, 50 mL), dried over MgSO_4_ and evaporated. The residue was crystallized from DIPE–hexane 3:1 solvent mixture to give 10.31 g (66%) of crude compound **3**. A small amount was recrystallized from DIPE for analytical purposes. Mp. 135–136.5 °C (DIPE). [α]_D_^25^ = −13° (c = 1.0, MeOH). IR (KBr, cm^−1^): $\tilde{\nu }$ 3397, 3102, 2980, 2934, 1697, 1538, 1508, 1457, 1387, 1355, 1295, 1253, 1231, 1205, 1164, 811, 755, 697. ^1^H NMR (400 MHz, DMSO-*d*_6_): *δ* 8.13 (br d, *J* = 8.4 Hz, 1H), 7.84 (~t, *J*_1_ = *J*_2_ = 8.4 Hz, 1H), 7.74 (dt, *J*_1_ = *J*_2_ = 8.4 Hz, *J*_3_ = 6.2 Hz, 1H), 7.56 (br m, 2H), 7.51 (br m, 1H), 7.39 (br, 2H), 7.21 (br d, *J* = 7.2 Hz, 1H), 4.06 (br, 1H), 1.68 (br, 1H), 1.44 (br, 1H), 1.37 (s, minor 9H), 1.34 (s, major 9H), 0.71 (br, 3H). ^13^C{^1^H} NMR (100 MHz, DMSO-*d*_6_): *δ* 175.37, 163.57, 157.21 (br d, *J* = 245 Hz), 155.86, 144.53 (br), 136.45 (br), 131.79 (br d, *J* ≈ 8.0 Hz), 129.96, 129.76, 128.87, 122.82 (d, *J* = 21.6 Hz), 120.75 (br), 78.53, 56.01 (br), 28.26, 23.39 (br), 10.86. (Figures S43–S45) Anal. calcd. for C_22_H_24_FN_3_O_6_ (445.45): C 59.32, H 5.43, N 9.43%; found: C 59.09, H 5.47, N 9.37%.

Synthesis of 5-fluoro-3-phenyl-2-[(1*R*)-1-aminopropyl]-4(3*H*)-quinazolinone hydrochloride (**6**)

A solution of compound **4** (8.24 g, 18.5 mmol) in THF (330 mL) was hydrogenated at atmospheric pressure in the presence of Pd/C catalyst (1.65 g). After removal of the catalyst, the solvent was evaporated to give compound **5** as a foam. Aqueous hydrochloride solution (10 w%, 76 mL) was added and it was refluxed for 30 min, then it was evaporated to dryness. Trituration with acetone gave 3.32 g (54%) of crystalline product **6**. [α]_D_^25^ = +35.4° (c = 1.0, MeOH). IR (KBr, cm^−1^): $\tilde{\nu }$ 3419, 2973, 2871, 1697, 1616, 1589, 1567, 1493, 1473, 1318, 1296, 1242, 1035, 822, 762, 702. ^1^H NMR (400 MHz, DMSO-*d*_6_): *δ* 8.87 (br, 3H), 7.92 (td, *J*_1_ = *J*_2_ = 8.4 Hz, *J*_3_ = 5.7 Hz), 7.72 (m, 1H), 7.66–7.54 (m, 5H), 7.39 (dd, *J*_1_ = 10.8 Hz, *J*_2_ = 8.4 Hz, 1H), 3.70 (m, 1H), 1.88 (m, 1H), 1.68 (m, 1H), 0.75 (t, *J*_1_ = *J*_2_ = 7.5 Hz, 3H). ^13^C{^1^H} NMR (100 MHz, DMSO-*d*_6_): *δ* 160.76 (d, *J* = 264 Hz), 158.14 (d, *J* = 4.3 Hz), 154.75, 148.35, 136.04 (d, *J* = 10.4 Hz), 135.58, 130.11, 129.93, 129.81, 129.78, 128.86, 123.34 (d, *J* = 3.7 Hz), 114.18 (d, *J* = 20.5 Hz), 110.68 (d, *J* = 5.9 Hz), 52.14, 25.27, 9.63. (Figures S46–S48) Anal. calcd. for C_17_H_17_ClFN_3_O (333.79): C 61.17, H 5.13, N 12.59, Cl 10.62%; found: C 61.19, H 5.36, N 12.05, Cl 10.23%.

Synthesis of 5-fluoro-3-phenyl-2-[(1*R*)-1-(7*H*-purin-6-ylamino)propyl]-4(3*H*)-quinazolinone (*R*-IDE, antipode of IDE)

A mixture of compound **6** (2.00 g, 6.00 mmol), 6-chloropurine (**7**, 1.02 g, 6.60 mmol) and DIPEA (2,52 mL, 1.82 g, 18.0 mmol) in *tert*-butanol (30 mL) was heated in an autoclave at 80 °C for 22 h. After evaporation of the solvent, the residue was triturated with water, filtered and crystallized from acetonitrile to give 1.02 g (41%) of product *R*-IDE. Mp. 259–262 °C. [α]_D_^25^ = −215.9° (c = 1.0, MeOH). IR (KBr, cm^−1^): $\tilde{\nu }$ 3235, 3159, 2977, 2764, 2597, 1689, 1629, 1591, 1567, 1473, 1453, 1328, 1299, 1253, 1231, 1160, 1033, 914, 819, 667, 616. ^1^H NMR (600 MHz, DMSO-*d*_6_): *δ* 12.99 (br, 1H), 8.17 (br s, 2H), 7.88 (br, 1H), 7.80 (td, *J*_1_ = *J*_2_ = 8.3 Hz, *J*_3_ = 5.9 Hz, 1H), 7.66, 7.59, 7.52 (br, 5H), 7.46 (d, *J* = 8.3 Hz, 1H), 7.28 (dd, *J*_1_ = 10.4 Hz, *J*_2_ = 8.3 Hz, 1H), 4.68 (br, 1H), 1.93 (br, 2H), 0.76 (br t, *J*_1_ = *J*_2_ = 7.1 Hz, 3H). ^13^C{^1^H} NMR (150 MHz, DMSO-*d*_6_): *δ* 160.76 (d, *J* = 264 Hz), 159.48 (br), 158.68 (br), 153.75 (br), 152.19, 149.99 (br), 149.31, 139.40, 136.30, 135.52 (d, *J* = 10.3 Hz), 129.49, 129.38, 129.23, 123.28, 119.10 (br), 113.31 (d, *J* = 20.4 Hz), 110.54 (d, *J* = 5.5 Hz), 53.84 (br), 25.8 (br), 11.20. (Figures S1–S3) Anal. calcd. for C_22_H_18_FN_7_O (415.42): C 63.61, H 4.37, N 23.60%; found: C 63.56, H 4.48, N 23.76%.

#### 3.2.2. Capillary Electrophoresis

CE analyses were performed using an Agilent HP 3D CE system (Agilent Technologies, Waldbronn, Germany) equipped with a photodiode array detector and controlled by ChemStation software (OpenLab CDS ChemStation Edition C.01.xx). Separations were carried out in untreated fused-silica capillaries (50 μm i.d., 48.5 cm total length, 40.0 cm effective length; Agilent).

New capillaries were conditioned by sequential rinsing with 1 M NaOH, 0.1 M NaOH, and deionized water, each for 30 min. Prior to each run, the capillary was preconditioned by flushing with the BGE for 5 min.

All analyses were performed at 25 °C with UV detection at 214 nm. Separation voltages were applied at 20–30 kV, and samples were injected hydrodynamically at 150 mbar·s. Investigated BGEs included citrate buffer (10–100 mM, pH 3.0) and phosphate buffer (20 mM, pH 2.5), adjusted with 1 M NaOH. CDs were added to the BGE at 10–30 mM. Stock solutions of IDE enantiomers (1 mg/mL in methanol) were diluted with the corresponding buffer for preliminary experiments.

For method validation, IDE sample solutions (5 mg/mL) were spiked with *R*-IDE stock solutions (0.10 and 0.25 mg/mL), all prepared in methanol. Design of experiments and statistical analyses were performed using Statistica v.14.0.0.15 (TIBCO Software Inc., Palo Alto, CA, USA), while validation data were evaluated in Microsoft Excel (Microsoft Office 365, Microsoft Corporation, Redmond, WA, USA).

#### 3.2.3. NMR Spectroscopy

NMR experiments were performed on Bruker Avance Neo 400 MHz and Bruker Avance III spectrometers (Bruker BioSpin, Billerica, MA, USA) operating at **^1^**H frequencies of 400, 500, 600, and 700 MHz. **^13^**C NMR spectra were acquired at 100, 125, and 150 MHz, while **^19^**F NMR experiments were conducted at a resonance frequency of 376 MHz. Standard pulse sequences from the TopSpin software (v. 3.6.2) library were employed for all experiments. For structure elucidation, IDE was first investigated in DMSO-*d*_6_ at a concentration of 40 mM. Variable-temperature measurements were carried out in the range of 298–363 K. Subsequently, IDE was studied in D_2_O at pD 3.0 (adjusted with 1 M HCl), at a concentration of 1 mM. The residual solvent signal and MeOH were used as a chemical shift scale reference. No external or internal reference compound was used for the ^19^F NMR measurements. CD complexation studies were performed in D_2_O at pD 3.0 using 1 mM IDE in the presence of β-CD or HP-β-CD (DS 6.8) at IDE:CD molar ratios of 1:1, 1:10, and 1:15. Non-equimolar mixtures of *R*- and *S*-enantiomer of IDE were prepared at a 1:4 molar ratio for enantiomer assignment. NOESY, ROESY and HOESY spectra were acquired with a mixing time of 350 ms and 700 ms, respectively.

##### Determination of the Activation Parameters

Variable-temperature ^1^H NMR spectra were recorded in DMSO-$d_{6}$ on a 500 MHz spectrometer over the temperature range of 298–363 K. The H18a, H18b, and H19 proton resonances were treated as an $A_{3}\mathrm{MNP}$ spin system (Figures S20–S26), and line-shape simulations were performed using gNMR v.5.0.6.0 to determine the exchange rate constants ($k_{\mathrm{exch}}$) associated with the hindered rotation (Table S14). The simulated spectra were iteratively fitted to the experimental spectra by optimizing the methyl and diastereotopic methylene resonances. The rate constants for the A → B ($k_{A\to B}$) and B → A ($k_{B\to A}$) atropisomerization processes were calculated from the exchange rate constant ($k_{\mathrm{exch}}$) obtained by line-shape simulation according to Equation (1),

(1) $$k=k_{A\to B}=k_{B\to A}=2\;\cdot \;k_{\mathrm{exch}}$$

assuming equal (50–50%) and temperature-independent populations of atropisomers A and B over the investigated temperature range.

The activation energy ($E_{a}$) was determined using the Arrhenius Equation (2):

(2) $$k\;=\;A\;\cdot \;\exp\left(–\frac{E_{a}}{\mathrm{RT}}\right)$$

where *A* is the pre-exponential factor, $E_{a}$ is the activation energy, *R* is the universal gas constant, and *T* is the absolute temperature.

The linearized form of the Arrhenius Equation (3):

(3) $$\ln\left(\frac{k}{s^{–1}}\right)\;=\;\ln\left(\frac{A}{s^{–1}}\right)-\frac{E_{a}}{R}\cdot \frac{1}{T}$$

was used by plotting $\ln\left(k\;/\;s^{–1}\right)$ versus 1/*T*. The activation energy was calculated from the slope of the least-squares linear regression.

The activation enthalpy ($∆H^{‡}$) and activation entropy ($∆S^{‡}$) were determined using the Eyring Equation (4):

(4) $$k\;=\;\frac{k_{B}T}{h}\exp\left(\frac{∆S^{‡}}{R}\right)\exp\left(–\frac{∆H^{‡}}{\mathrm{RT}}\right)$$

where $k_{B}$ is the Boltzmann constant, *h* is the Planck constant, *R* is the universal gas constant, and *T* is the absolute temperature.

The linearized form of the Eyring Equation (5) is given by:

(5) $$\ln\left(\frac{k\;/\;T}{s^{–1}\;K^{–1}}\right)\;=\;\ln\left(\frac{k_{B}\;/\;h}{s^{–1}\;K^{–1}}\right)\;+\;\frac{∆S^{‡}}{R}-\frac{∆H^{‡}}{R}\cdot \frac{1}{T}$$

Linear regression of $\ln[(k\;/\;T)\;/\;(s^{–1}\;K^{–1})]$ versus 1/*T* was performed using the least-squares method. The activation enthalpy ($∆H^{‡}$) and activation entropy ($∆S^{‡}$) were calculated from the slope and intercept of the fitted line, respectively. Finally, the Gibbs free energy of activation ($∆G^{‡}$) at *T* = 298.15 K was calculated according to Equation (6)

(6) $$∆G^{‡}\;=∆H^{‡}-T∆S^{‡}$$

The confidence intervals of the fitted parameters corresponded to the 95% confidence level.

## 4. Conclusions

A robust and sensitive CD-mediated CE method was developed and validated for trace-level quantification of the *R*-enantiomer of IDE, addressing the methodological gap in its detection at trace levels. Systematic selector screening identified HP-β-CD with intermediate DS (~6.8) as optimal, suggesting a favourable balance between cavity accessibility and conformational flexibility for effective enantioseparation. DoE-based optimization ensured robust operation with maximal resolution and acceptable peak symmetry.

Mechanistic insights from NMR experiments revealed a hindered *C*-*N* rotation in IDE, while complexation with β-CD and HP-β-CD stabilized specific conformational states of IDE and demonstrated distinct binding behaviours for the *S*- and *R*-enantiomers. Both ^1^H and ^19^F NMR revealed enantiomeric discrimination, with ^19^F spectra providing simplified, less congested signals and distinct exchange regimes. Consequently, ^19^F NMR proved a sensitive probe and offered mechanistic insight into the host-guest interactions.

2D NMR experiments (NOESY, ROESY, and HOESY) confirmed the inclusion of the fluorinated aromatic moiety within the CD cavity. Compared with β-CD, HP-β-CD exhibited a more extensive interaction pattern, consistent with multiple host–guest binding modes and the coexistence of 1:1 and 1:2 complexes in solution. This structural feature explains the enhanced enantioselectivity observed in CE and provides a mechanistic basis for CD-mediated molecular recognition.

Although developed for IDE, the combined CE-DoE-NMR approach offers a generalizable strategy for the analysis of chiral, fluorinated 3-phenylquinazolin-4-one-based kinase inhibitors. This strategy may be extended to structurally related fluorinated APIs, supporting both quality control and mechanistic studies in pharmaceutical analysis.

## Figures and Tables

**Table 1:** Method validation data for the quantification of enantiomeric impurity *R*-IDE (20 mM HP-β-CD (DS~6.8), 25 mM citrate buffer (pH 3.0), applied voltage 25 kV, temperature 25 °C).

Parameter	Concentration (µg/mL)	*R*-IDE
LOD	-	2 µg/mL
LOQ	-	5 µg/mL (0.1%)
Range (µg/mL)	-	5–25 (0.1–0.5%)
Equation	-	*y* = 1.64618*x* + 0.833396
*R* ^2^	-	0.99916
Accuracy (%) ^1^	5	101.64 ± 6.43
	15	100.30 ± 3.06
	25	101.97 ± 2.71
Intraday precision (RSD%) ^1^	5	3.75
	15	3.77
	25	3.36
Intermediate precision	5	3.94
(interday) (RSD%) ^1^	15	5.91
	25	3.59

^1^ Peak area.

## Data Availability

The original contributions presented in this study are included in the article/Supplementary Material. Further inquiries can be directed to the corresponding authors.

## Supplementary Materials

The following supporting information can be downloaded at: https://www.mdpi.com/article/10.3390/ijms27136036/s1.

## Abbreviations

API	Active Pharmaceutical Ingredient
BGE	Background Electrolyte
β-CD	β-Cyclodextrin
CD	Cyclodextrin
CE	Capillary Electrophoresis
COSY	Correlation Spectroscopy
DIPE	Diisopropyl Ether
DIPEA	*N*,*N*-diisopropylethylamine
DMF	Dimethylformamide
DMSO	Dimethyl Sulfoxide
DoE	Design of Experiments
DS	Degree of Substitution
ee	Enantiomeric Excess
EMO	Enantiomer Migration Order
HMBC	Heteronuclear Multiple Bond Correlation
HOESY	Heteronuclear Overhauser Effect Spectroscopy
HPLC	High-Performance Liquid Chromatography
HP-β-CD	Hydroxypropyl-β-Cyclodextrin
HSQC	Heteronuclear Single Quantum Coherence
ICH	International Council for Harmonisation
IDE	Idelalisib
LOQ	Limit of Quantification
LOD	Limit of Detection
MeOH	Methanol
NMR	Nuclear Magnetic Resonance
NOESY	Nuclear Overhauser Effect Spectroscopy
pD	pH Measured in Deuterated Solvent
PI3Kδ	Phosphoinositide 3-Kinase Delta
*R*-IDE	*R*-Enantiomer of Idelalisib
ROESY	Rotating-frame Overhauser Effect Spectroscopy
*R_s_*	Resolution
RSD	Relative Standard Deviation
*S*-IDE	*S*-Enantiomer of Idelalisib
TGA	Therapeutic Goods Administration
UV	Ultraviolet
