# Supplementary material for: Cyclodextrin-Mediated Enantiomeric Separation of Idelalisib: A Validated Capillary Electrophoresis and NMR Study
Source: Int J Mol Sci. 2026 Jul 5;27(13):6036. doi: 10.3390/ijms27136036 (PMC13360804; doi:10.3390/ijms27136036)
Supplement: Supplementary file 1 [file ijms-27-06036-s001.zip › ijms-4331856-updated SM.pdf]

Cyclodextrin-Mediated Enantiomeric Separation of Idelalisib: A Validated Capillary Electrophoresis and NMR Study

Erzsébet Várnagy [1,2], Balázs István Urbán [3], Mátyás Sári [3], Balázs Volk [4], Gyula Simig [4], Krisztina Németh [5], Milo Malanga [6], Ida Fejős [1,2]*, Szabolcs Béni [3]*

1 Department of Pharmacognosy, Semmelweis University, Üllői út 26, H-1085 Budapest, Hungary
2 Center for Pharmacology and Drug Research & Development, Semmelweis University, Üllői út 26, H-1085 Budapest, Hungary
3 Integrative Health and Environmental Analysis Research Laboratory, Department of Analytical Chemistry, Institute of Chemistry, ELTE Eötvös Loránd University, Pázmány Péter sétány 1/A, H-1117 Budapest, Hungary
4 Directorate of Drug Substance Development, Egis Pharmaceuticals Plc., P.O. Box 100, H-1475 Budapest, Hungary
5 Chemical Biology Research Group, Institute of Organic Chemistry, HUN-REN Research Centre for Natural Sciences, Magyar Tudósok Krt. 2., 1117 Budapest, Hungary
6 CarboHyde Ltd., Berlini u. 47-49, H-1045 Budapest, Hungary

* Correspondence: szabolcs.beni@ttk.elte.hu (S.B.) and fejos.ida@semmelweis.hu (I.F.)

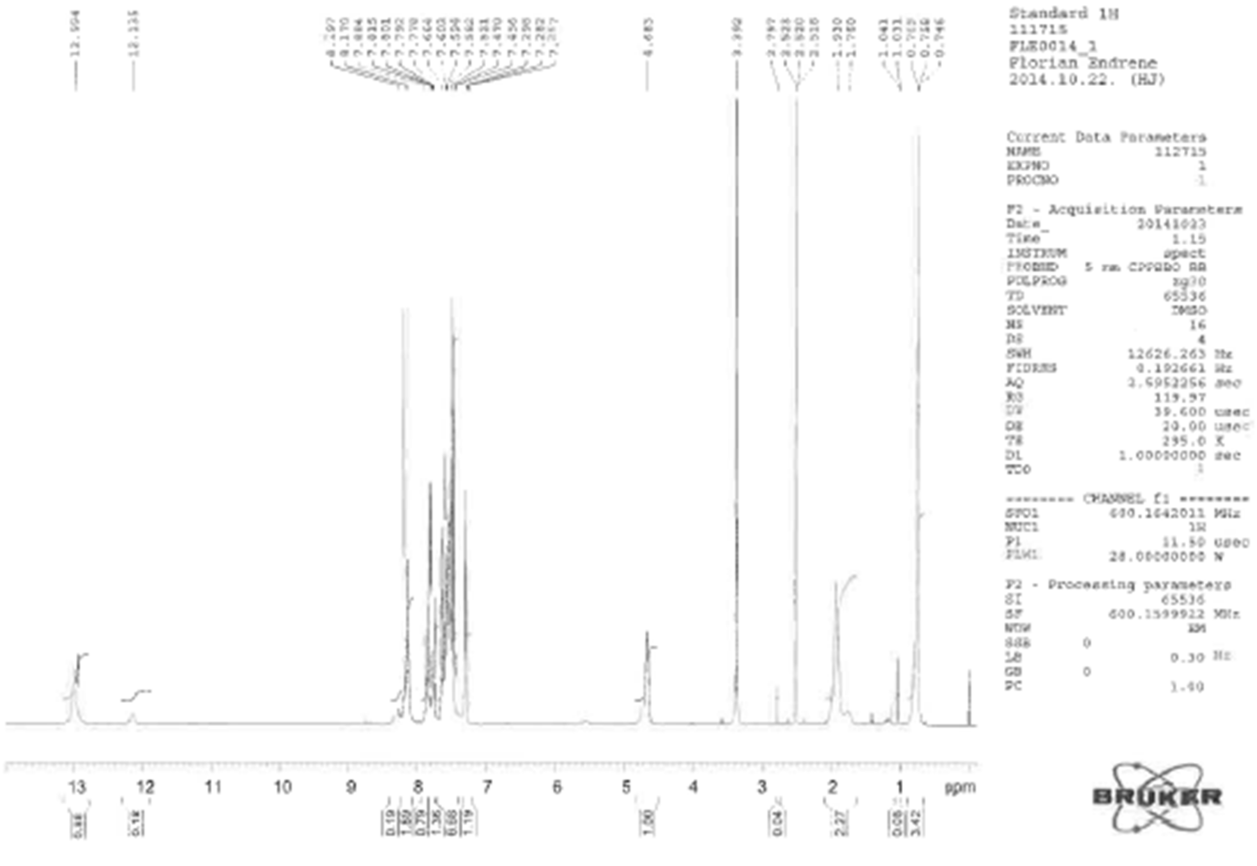

Figure S1. ^{1}H NMR spectrum of *R*-IDE.

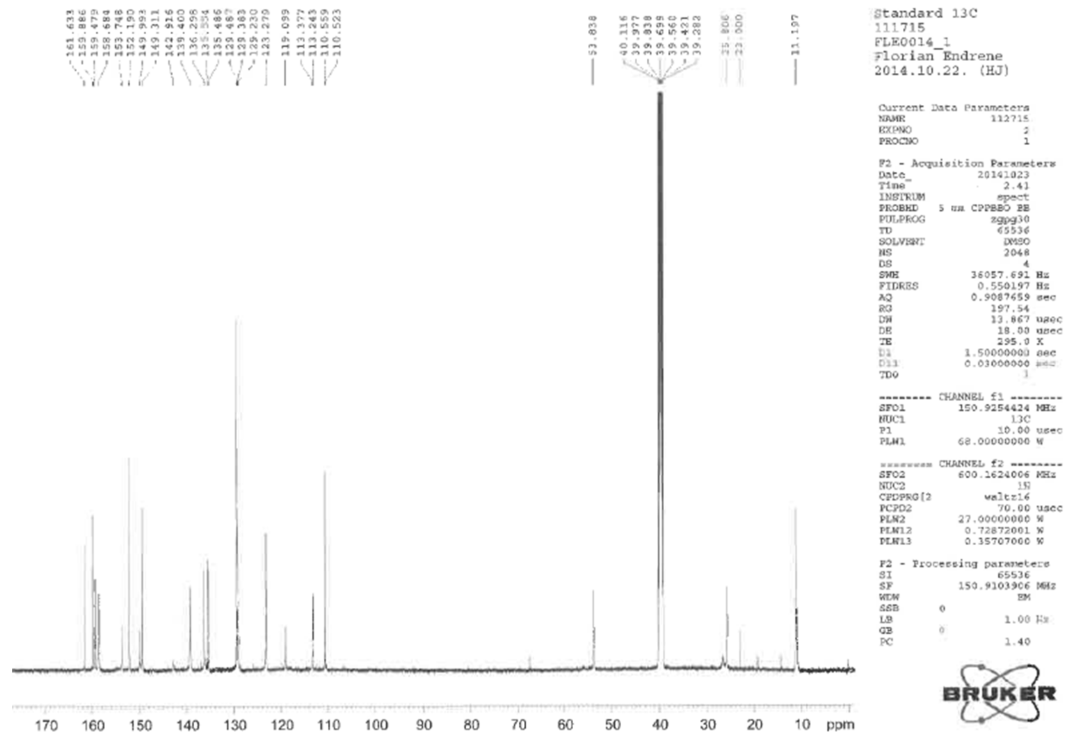

Figure S2. ^{13}C NMR spectrum of compound *R*-IDE.

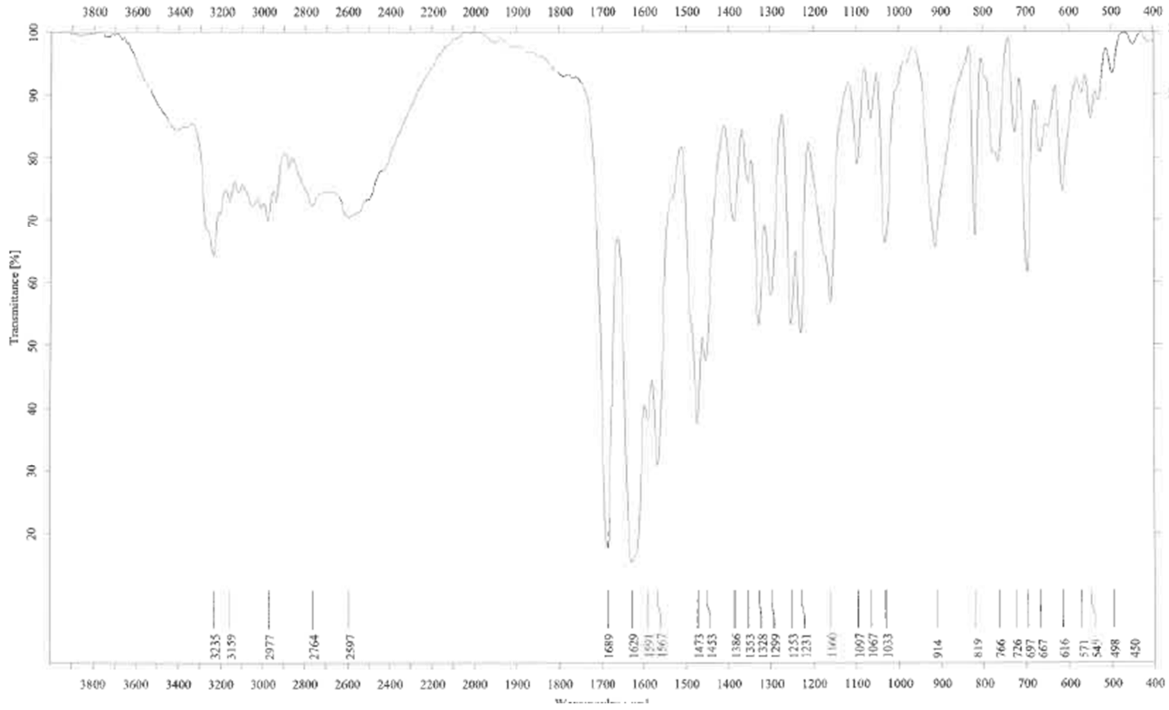

Figure S3. IR spectrum of compound *R*-IDE.

Table S1. Independent variables (factors) and their coded and actual levels used in the Box-Behnken experimental design.

Factor	Low (-1)	Medium (0)	High (+1)
Voltage (kV)	20	25	30
CD concentration (mM)	10	20	30
Buffer concentration (mM)	10	25	50

Table S2. Experimental results of the Box-Behnken design for resolution (R_s) and distomer peak symmetry, the runs were in randomized order.

#	Voltage [kV]	CD conc. [mM]	Buffer conc. [mM]	R_s	Symmetry of the distomer
1	20	10	25	3.39	1.488
2	30	10	25	3.10	1.380
3	20	30	25	3.42	1.230
4	30	30	25	4.07	1.100
5	25	10	10	2.81	1.020
6	25	30	10	3.10	1.039
7	25	10	50	2.83	2.970
8	25	30	50	3.66	1.370
9	20	20	10	1.65	0.735
10	30	20	10	1.49	1.100
11	20	20	50	3.83	1.960
12	30	20	50	4.36	2.320
13	25	20	25	4.15	1.290
14	25	20	25	4.02	0.955
15	25	20	25	4.36	1.120

Table S3. Statistical analysis of the Box-Behnken design for resolution (R_s).

Factor	Effect Estimates; Var.:Resolution; R-sqr=,99466; Adj:,9626 (idelalisib-CE-250529)<br>3 3-level factors, 1 Blocks, 15 Runs; MS Residual=,0294333<br>DV: Resolution									
	Effect	Std.Err.	t(2)	p	-95,% Cnf.Limt	+95,% Cnf.Limt	Coeff.	Std.Err. Coeff.	-95,% Cnf.Limt	+95,% Cnf.Limt
Mean/Interc.	3,24771	0,050039	64,90386	0,000237	3,03241	3,463008	3,247708	0,050039	3,032409	3,463008
(1)Voltage(L)	0,23917	0,131032	1,82525	0,209510	-0,32462	0,802953	0,119583	0,065516	-0,162310	0,401476
Voltage(Q)	0,33490	0,090562	3,69797	0,065972	-0,05476	0,724553	0,167448	0,045281	-0,027380	0,362276
(2)CD conc.(L)	0,56500	0,131032	4,31192	0,049801	0,00121	1,128786	0,282500	0,065516	0,000607	0,564393
CD conc.(Q)	0,06740	0,090562	0,74420	0,534316	-0,32226	0,457053	0,033698	0,045281	-0,161130	0,228526
(3)Buffer conc.(L)	1,78000	0,127874	13,91991	0,005121	1,22980	2,330199	0,890000	0,063937	0,614901	1,165099
Buffer conc.(Q)	1,04552	0,090562	11,54481	0,007419	0,65586	1,435178	0,522760	0,045281	0,327932	0,717589
1L by 2L	0,47000	0,171561	2,73954	0,111413	-0,26817	1,208169	0,235000	0,085781	-0,134085	0,604085
1L by 2Q	-0,04062	0,123193	-0,32977	0,772912	-0,57068	0,489433	-0,020312	0,061597	-0,285341	0,244716
1Q by 2L	-0,00375	0,123193	-0,03044	0,978481	-0,53381	0,526308	-0,001875	0,061597	-0,266904	0,263154
1L by 3L	0,34500	0,171561	2,01094	0,182023	-0,39317	1,083169	0,172500	0,085781	-0,196585	0,541585
1Q by 3L	-1,11750	0,121312	-9,21176	0,011580	-1,63946	-0,595535	-0,558750	0,060656	-0,819732	-0,297768
2L by 3L	0,27000	0,171561	1,57378	0,256192	-0,46817	1,008169	0,135000	0,085781	-0,234085	0,504085

Table S4. Statistical analysis of the Box-Behnken design for peak symmetry of the distomer.

Factor	Effect	Std.Err.	t(2)	p	-95,% Cnf.Limt	+95,% Cnf.Limt	Coeff.	Std.Err. Coeff.	-95,% Cnf.Limt	+95,% Cnf.Limt
	Effect Estimates; Var.Symmetry1; R-sqr=,98842; Adj:,91897 (idelalisib-CE-250529) 3 3-level factors, 1 Blocks, 15 Runs; MS Residual=,0280583 DV: Symmetry1									
Mean/Interc.	1,526938	0,048856	31,25385	0,001022	1,31673	1,737148	1,526938	0,048856	1,316727	1,737148
(1)Voltage(L)	0,041083	0,127935	0,32113	0,778566	-0,50938	0,591543	0,020542	0,063967	-0,254688	0,295772
Voltage(Q)	-0,058542	0,088421	-0,66208	0,576005	-0,43899	0,321905	-0,029271	0,044211	-0,219494	0,160952
(2)CD conc.(L)	-0,577750	0,127935	-4,51597	0,045699	-1,12821	-0,027290	-0,288875	0,063967	-0,564105	-0,013645
CD conc.(Q)	-0,129542	0,088421	-1,46505	0,280521	-0,50999	0,250905	-0,064771	0,044211	-0,254994	0,125452
(3)Buffer conc(L)	1,195167	0,124852	9,57269	0,010737	0,65797	1,732360	0,597583	0,062426	0,328986	0,866180
Buffer conc(Q)	-0,205979	0,088421	-2,32952	0,145190	-0,58643	0,174467	-0,102990	0,044211	-0,293213	0,087234
1L by 2L	-0,011000	0,167506	-0,06567	0,953615	-0,73172	0,709721	-0,005500	0,083753	-0,365861	0,354861
1L by 2Q	0,241063	0,120281	2,00416	0,182939	-0,27647	0,758591	0,120531	0,060141	-0,138233	0,379295
1Q by 2L	-0,159563	0,120281	-1,32658	0,315852	-0,67709	0,357966	-0,079781	0,060141	-0,338545	0,178983
1L by 3L	-0,002500	0,167506	-0,01492	0,989447	-0,72322	0,718221	-0,001250	0,083753	-0,361611	0,359111
1Q by 3L	-0,041000	0,118445	-0,34615	0,762251	-0,55063	0,468627	-0,020500	0,059222	-0,275313	0,234313
2L by 3L	-0,809500	0,167506	-4,83266	0,040251	-1,53022	-0,088779	-0,404750	0,083753	-0,765111	-0,044389

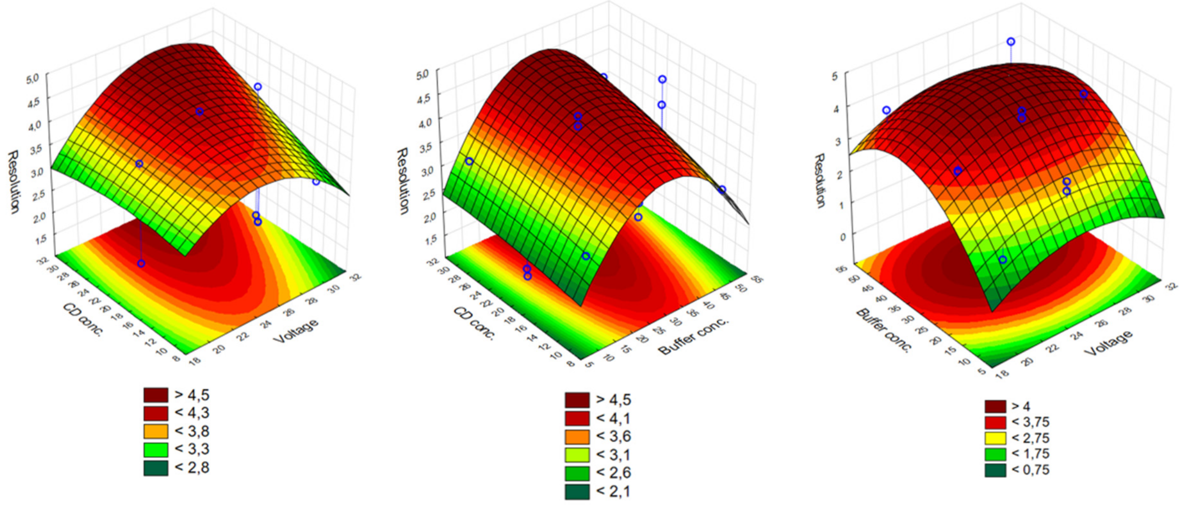

Figure S4. Box-Behnken surface plots (variable: Resolution).

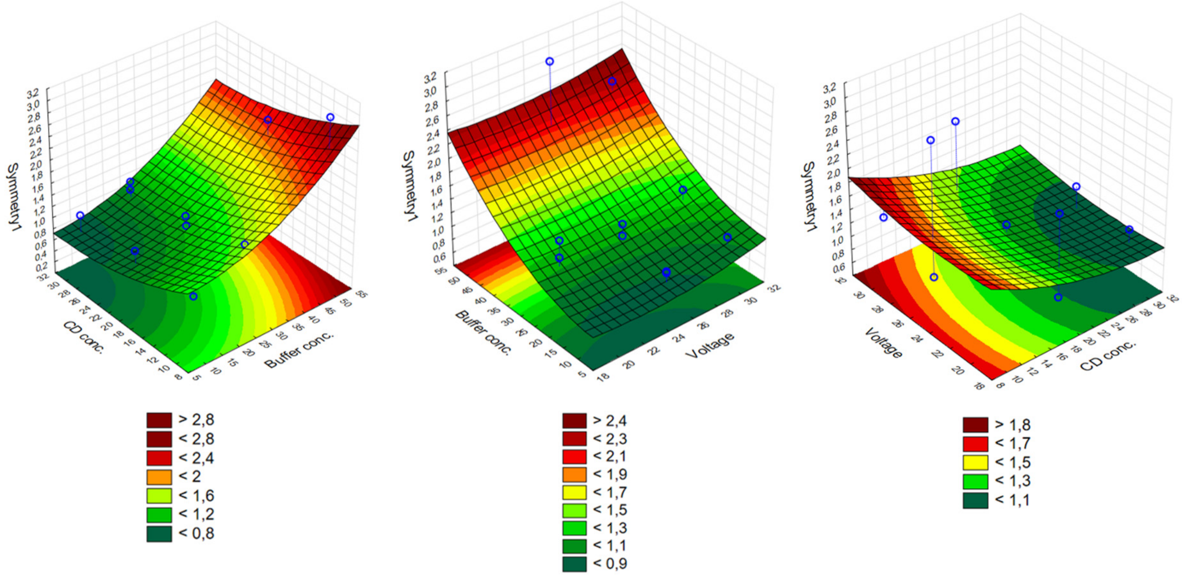

Figure S5. Box-Behnken surface plots (variable: peak symmetry of the distomer).

Table S5. Independent variables (factors) and their coded and actual levels used in the two-level screening Plackett-Burman design for robustness testing.

Factor	Low (-)	Centre (0)	High (+)
CD concentration (mM)	19	20	21
Buffer concentration (mM)	24	25	26
Voltage (kV)	24	25	26
pH	2.9	3.0	3.1
Temperature (°C)	24	25	26

Table S6. Design matrix of the Plackett-Burman design used for robustness testing. The runs were in randomized order. The dependent variable was the resolution (R_s).

#	HP-β-CD (mM)	Buffer (mM)	Voltage (kV)	pH	Temperature (°C)	R_s	Symmetry of the distomer
1	21	24	24	3.1	24	4.20	1.06
2	21	26	24	2.9	26	4.20	1.06
3	21	26	26	2.9	24	4.14	1.06
4	19	26	26	3.1	24	3.94	1.06
5	21	24	26	3.1	26	3.99	1.06
6	19	26	24	3.1	26	4.20	1.06
7	19	24	26	2.9	26	3.81	1.06
8	19	24	24	2.9	24	4.00	1.06
9	20	25	25	3.0	25	4.30	1.06
10	20	25	25	3.0	25	4.10	1.06
11	20	25	25	3.0	25	4.10	1.06

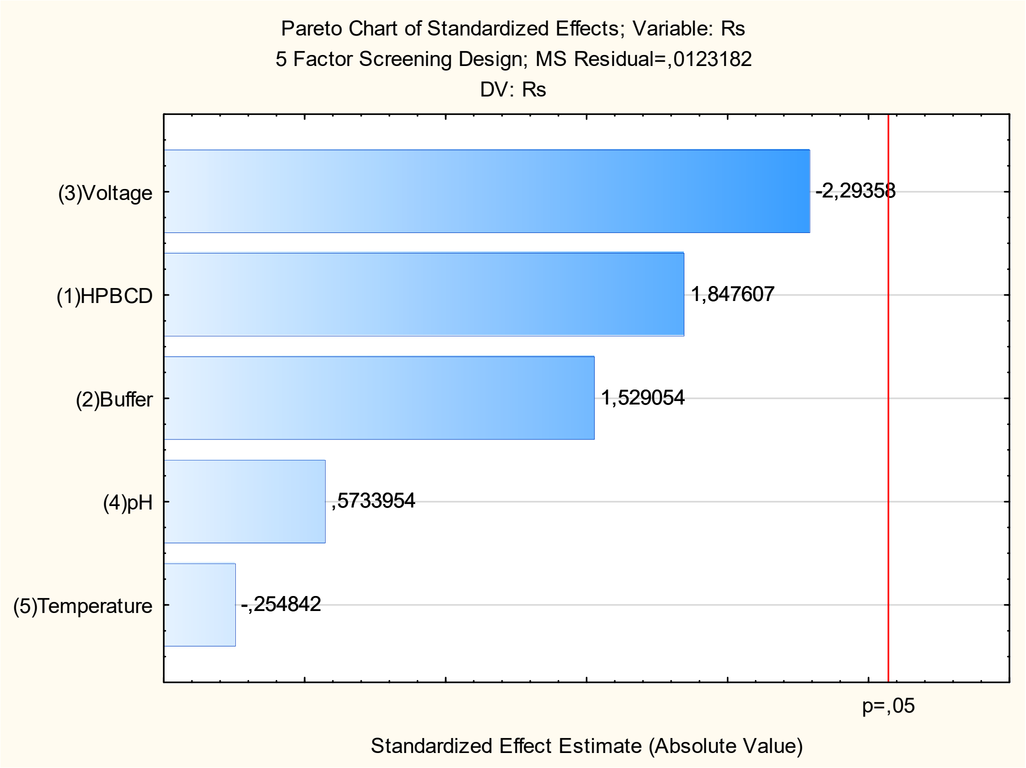

Figure S6. Pareto chart of standardized effects using Statistica software. Dependent variable: resolution.

Table S7. Validation data – linearity.

R-IDE conc. (µg/ml)	Area1	Area2	Area3	Area4	Area5	Mean	Std. deviation	RSD%
**5**	9.00	9.50	8.60	9.30	9.20	9.12	0.34	3.75
**10**	15.4	16.0	17.2	17.6	17.1	16.66	0.92	5.53
**15**	24.0	26.3	25.8	25.7	26.4	25.64	0.97	3.77
**20**	35.0	34.8	34.9	34.7	31.1	34.10	1.68	4.93
**25**	41.8	41.5	44.4	40.6	42.0	42.06	1.41	3.36

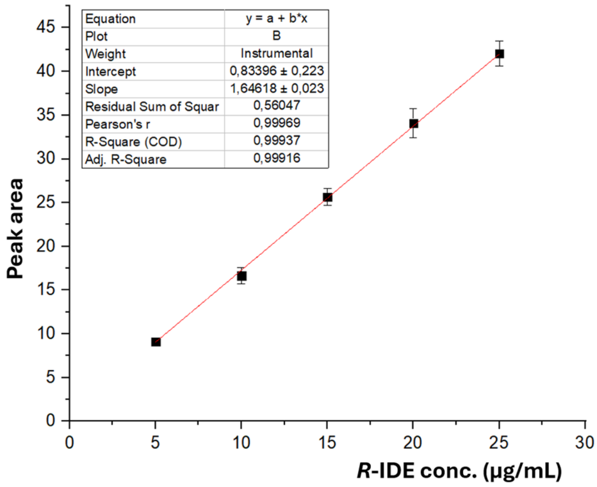

Figure S7. Calibration solution of *R*-idelalisib (*R*-IDE) ranged between 5-25 µg/mL.

Table S8. Validation data – accuracy.

R-IDE conc. (µg/ml)	Area1	Area2	Area3	Recovery1	Recovery2	Recovery3	Mean	Std. deviation
**5**	8.6	9.4	9.6	94.35	104.07	106.50	101.64	6.43
**15**	25.7	26.3	24.8	100.70	103.13	97.06	100.30	3.06
**25**	41.8	44.0	42.6	99.54	104.89	101.49	101.97	2.71

Table S9. Validation data – repeatability, intraday precision.

R-IDE conc. (µg/ml)	Area1	Area2	Area3	Area4	Area5	Mean	Std. deviation	RSD%
**5**	9.00	9.50	8.60	9.30	9.20	9.12	0.34	3.75
**15**	24.0	26.3	25.8	25.7	26.4	25.64	0.97	3.77
**25**	41.8	41.5	44.4	40.6	42.0	42.06	1.41	3.36

Table S10. Validation data – repeatability, intermediate precision.

	Day1					Day2					Intermediate precision		
R-IDE conc. (ug/ml)	Area1	Area2	Area3	Area 4	Area 5	Area 1	Area 2	Area 3	Area 4	Area 5	Mean	Std. dev.	RSD %
**5**	9	8.6	9.3	9.2	8.6	8.9	8.5	9.4	8.9	9.5	8.99	0.35	3.94
**15**	24	25.8	25.7	25.7	24.8	25.1	22.7	22.7	23.1	22.1	24.17	1.43	5.91
**25**	41.8	41.5	40.6	41.8	42.6	40.9	40.3	38.8	39.1	38.2	40.56	1.46	3.59

Table S11. Validation data, intermediate precision - migration times of *R*-IDE (5 μg/mL).

Day	Run	Migration time	Mean [min]	SD [min]	RSD%
Day1	1	7.473	7.54	0.17	2.30
	2	7.774			
	3	7.733			
	4	7.511			
	5	7.336			
	6	7.433			
Day2	1	9.075	8.74	0.31	3.58
	2	9.007			
	3	8.92			
	4	8.663			
	5	8.445			
	6	8.314			
Day3	1	8.151	7.90	0.19	2.42
	2	8.025			
	3	7.995			
	4	7.839			
	5	7.750			
	6	7.637			
Intermediate precision (n=18)			8.06	0.56	6.96

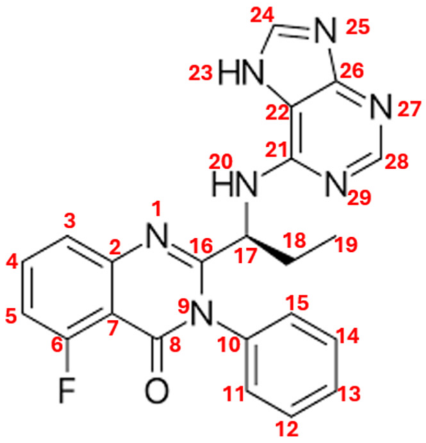

Figure S8. The structure of idelalisib (*S*-IDE) with atom numbering.

Table S12. ^{1}H and ^{13}C assignment of idelalisib (*S*-IDE)**,** (in DMSO-d6; 298 K; 500 MHz). For the atomic positions of (*S*-IDE) see Figure S8.

**Atomic position**	**^{1}H δ (ppm)**	**^{13}C δ (ppm)**
1	-	-
2	-	149.6
3	7.47 (d, J=8.2 Hz; 1H)	123.6
4	7.80 (td, J= 8.0; 6.0 Hz; 1H)	135.8 (d, J= 10 Hz)
5	7.28 (~t, broad, 1H)	113.6 (d, J= 20 Hz)
6	-	159.8 (d, J= 34.5 Hz)
7	-	110.8 (d, J= 5.0 Hz)
8	-	162.1
9	-	-
10	-	136.5
11-15	7.49-7.68 (m, broad, 6H)	129.7
16a	-	158.9
16b	-	158.8
17	4.68 (s, broad, 1H)	54.1
18	1.93 (m, broad, 2H)	26.1
19	0.76 (t, broad, 3H)	11.4
20a	7.75 (m, broad)	-
20b	7.85 (m, broad)	-
21	-	150.3
22	-	119.4
23a	12.97 (s, 1H)	-
23b	12.10 (s, broad)	-
24a	8.16 (s, 1H)	139.6
24b	8.29	143.1
25	-	-
26	-	154.0
27	-	-
28a	8.16 (s, 1H)	152.4
28b	8.21	152.4
29	-	-

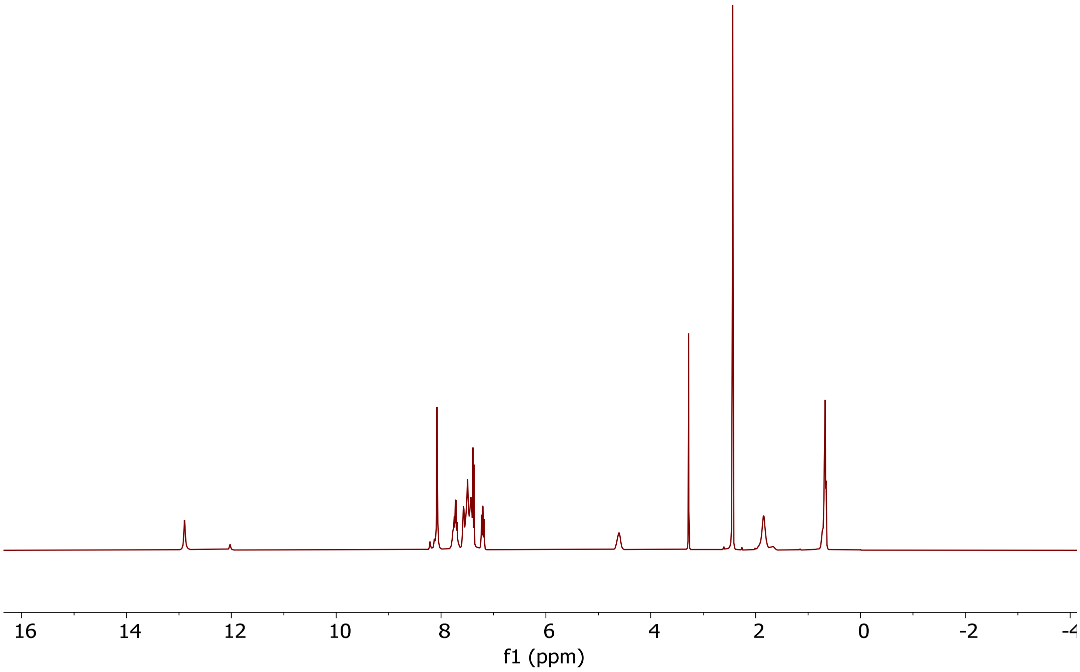

Figure S9. ^{1}H NMR spectrum of idelalisib (*S*-IDE), (in DMSO-d_6; 298 K; 400 MHz).

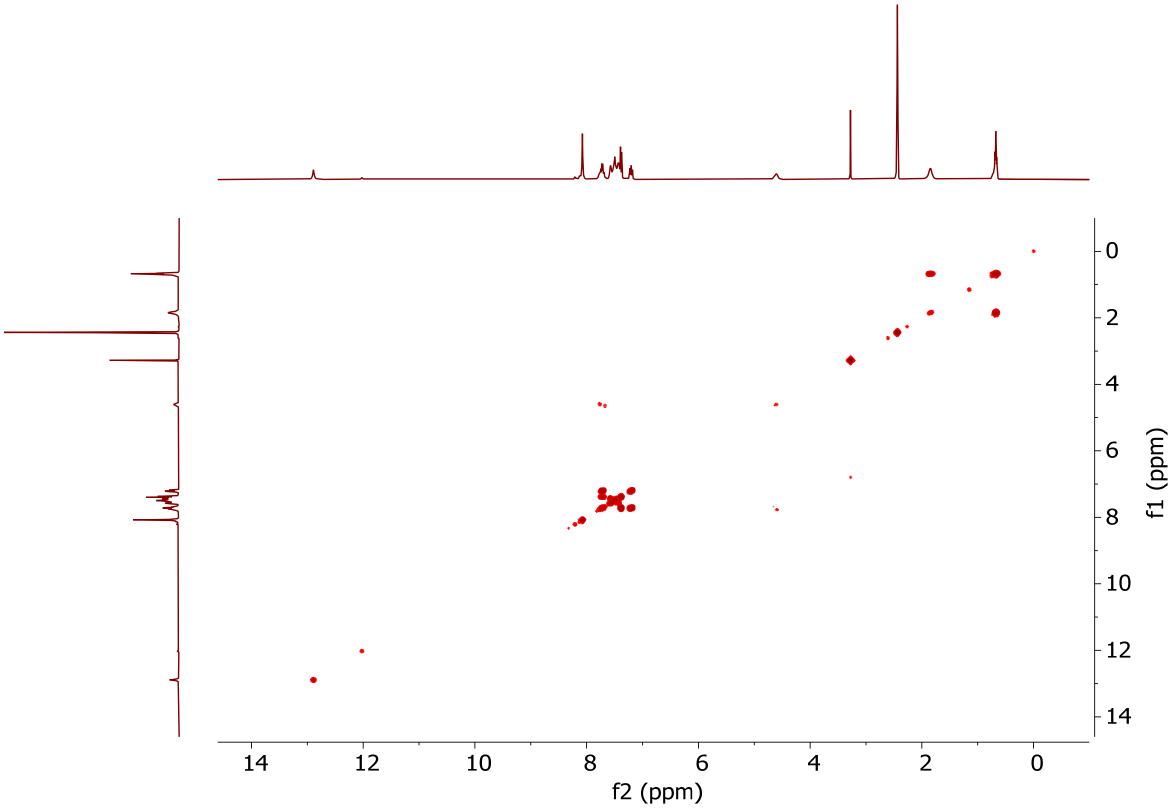

Figure S10. COSY spectrum of idelalisib (*S*-IDE), (in DMSO-d_6; 298 K; 400 MHz).

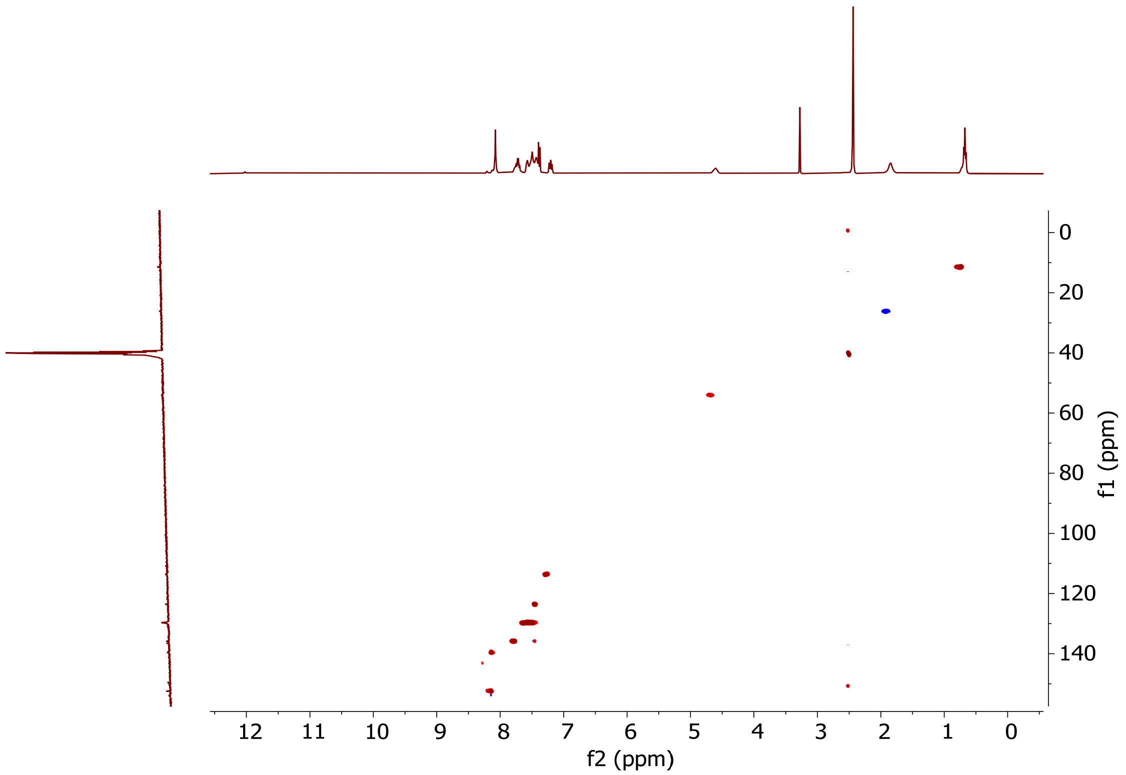

Figure S11. HSQC spectrum of idelalisib (*S*-IDE), (in DMSO-d6; 298 K; 400 MHz).

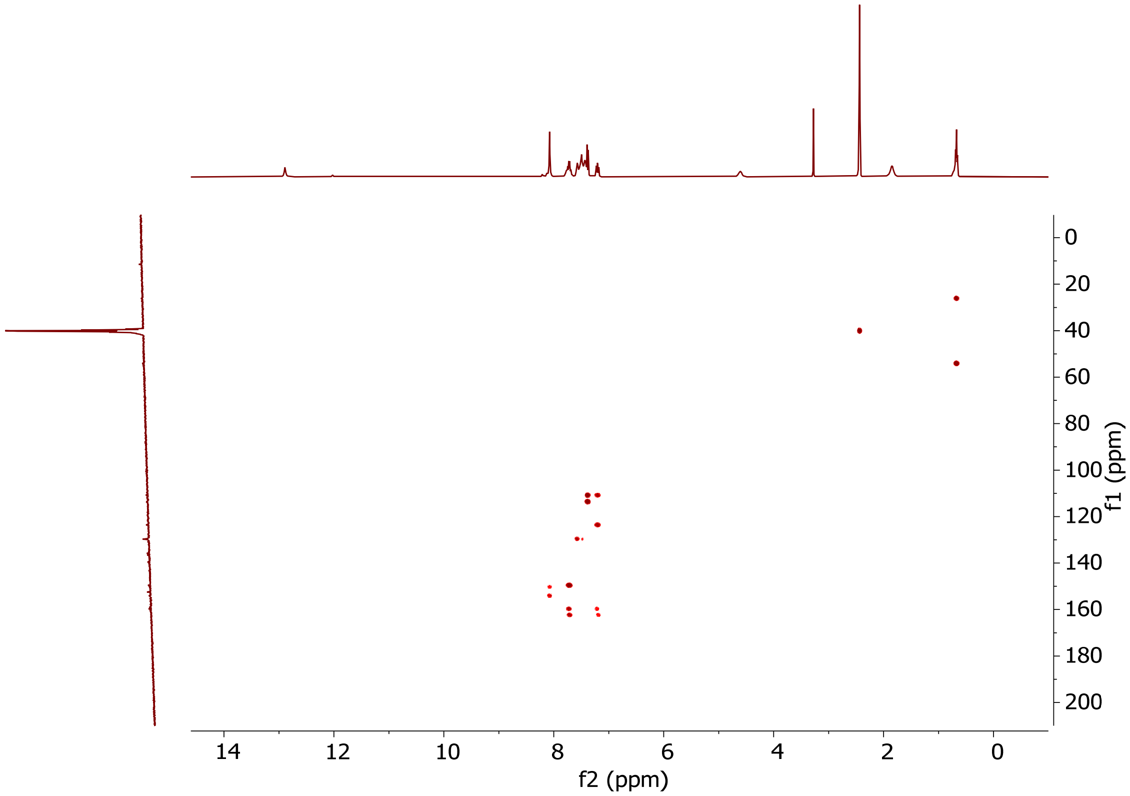

Figure S12. HMBC spectrum of idelalisib (*S*-IDE), (in DMSO-d6; 298 K; 400 MHz).

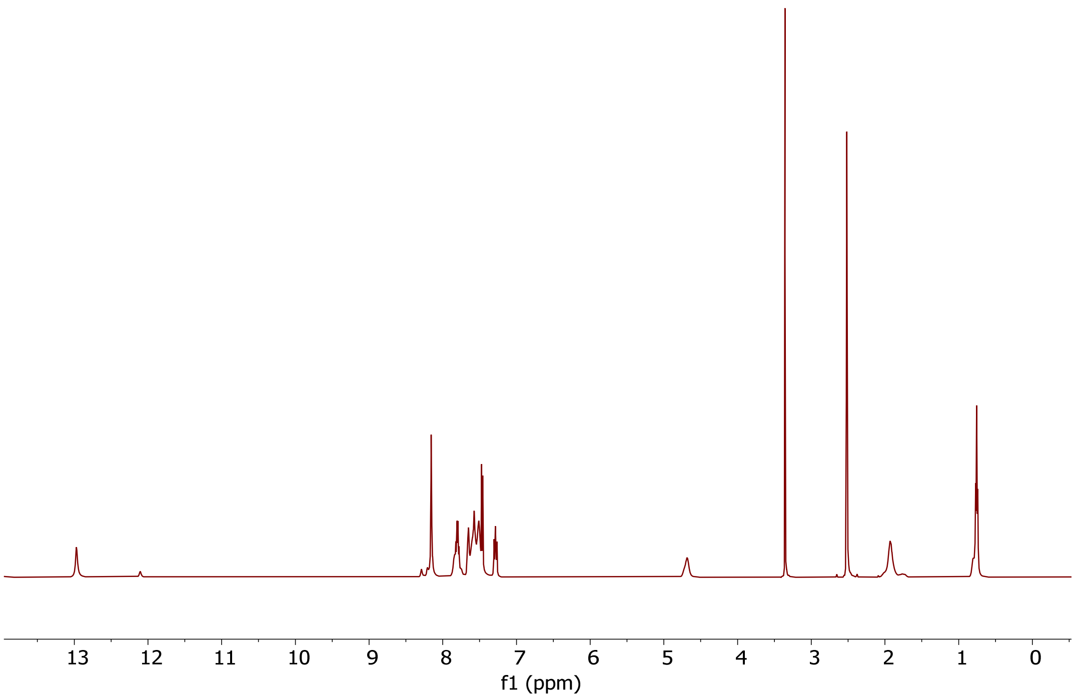

Figure S13. ^{1}H NMR spectrum of idelalisib (*S*-IDE), (in DMSO-d_6; 298 K; 500 MHz).

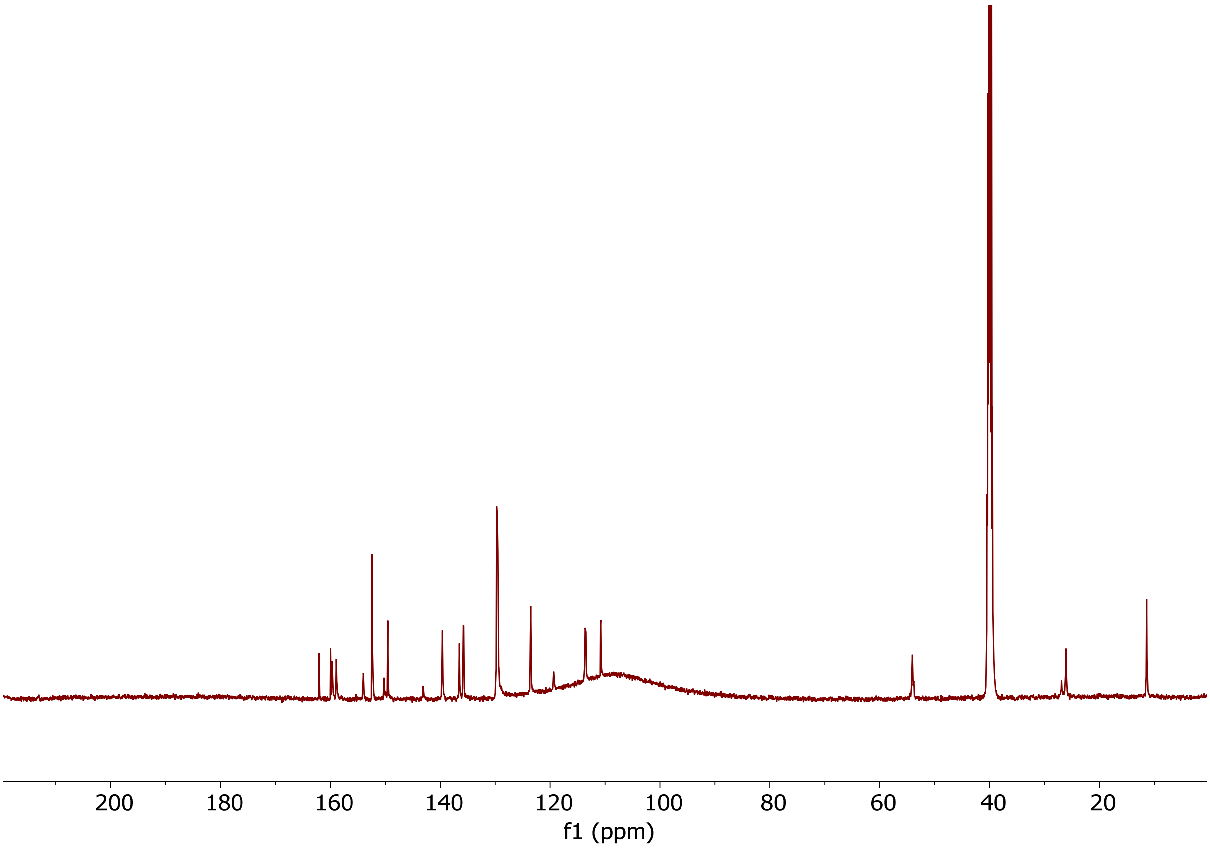

Figure S14. ^{13}C NMR spectrum of idelalisib (*S*-IDE), (in DMSO-d_6; 298 K; 125 MHz).

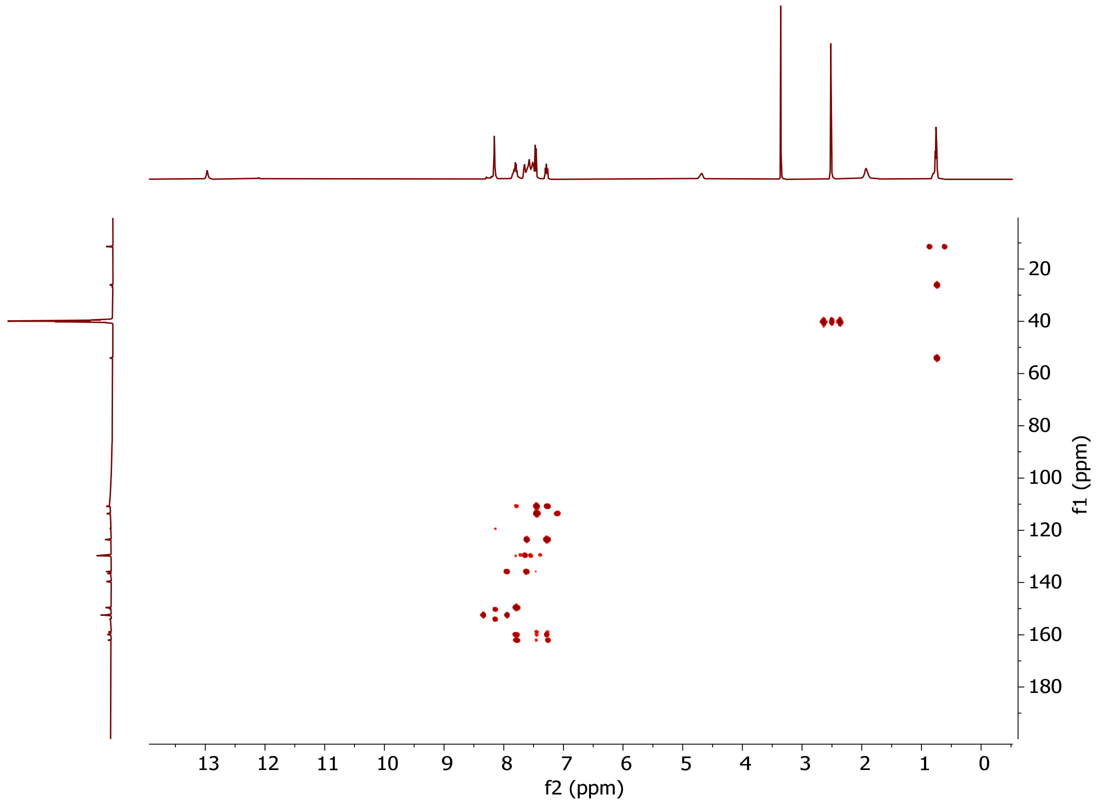

Figure S15. HMBC spectrum of idelalisib (*S*-IDE), (in DMSO-d6; 298 K; 500 MHz).

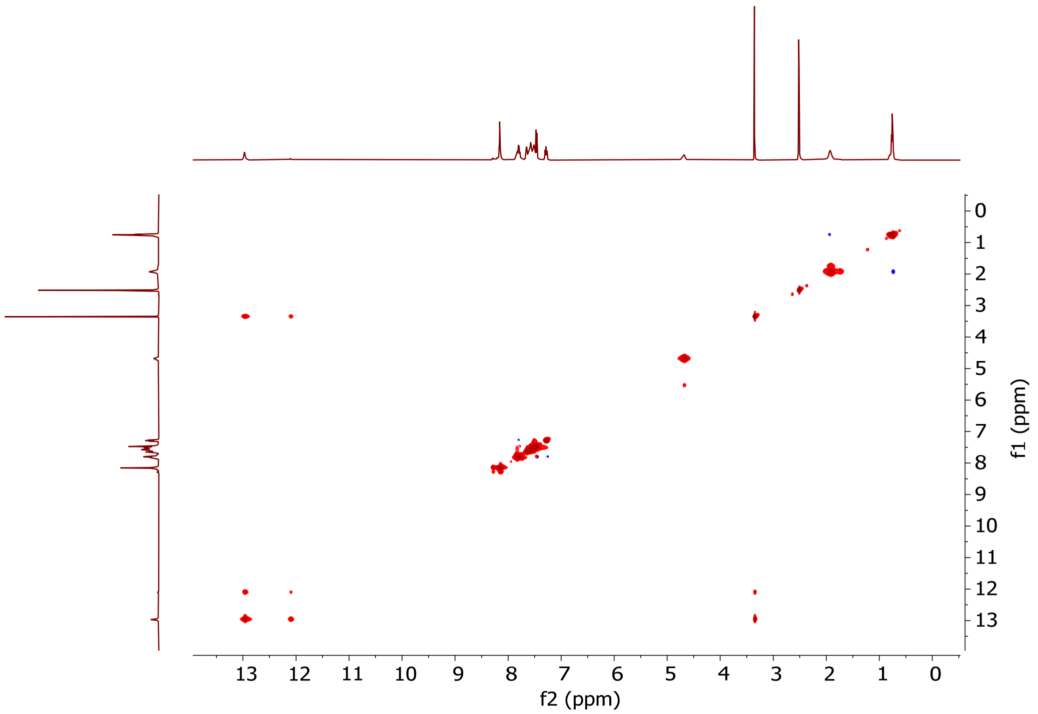

Figure S16. NOESY spectrum of idelalisib (*S*-IDE), (in DMSO-d6; 298 K; 500 MHz).

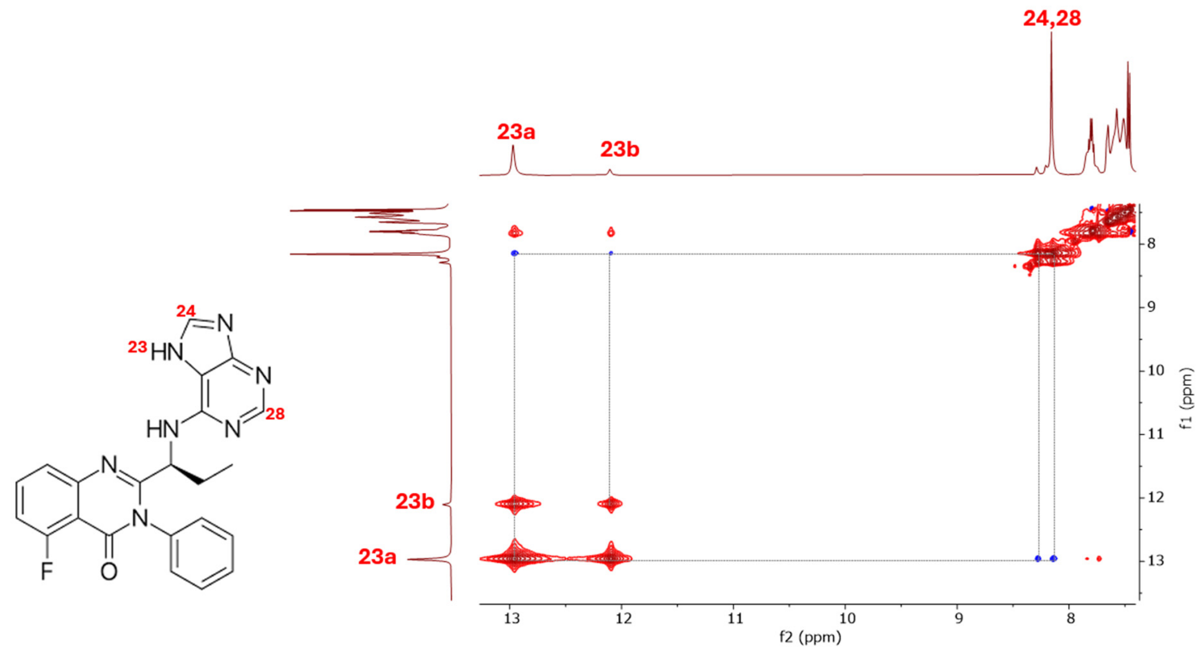

Figure S17. Chemical structure of *S*-IDE, with atom numbering (left). Partial NOESY NMR spectrum of IDE recorded at 500 MHz in DMSO-d_6 at 298 K (right).

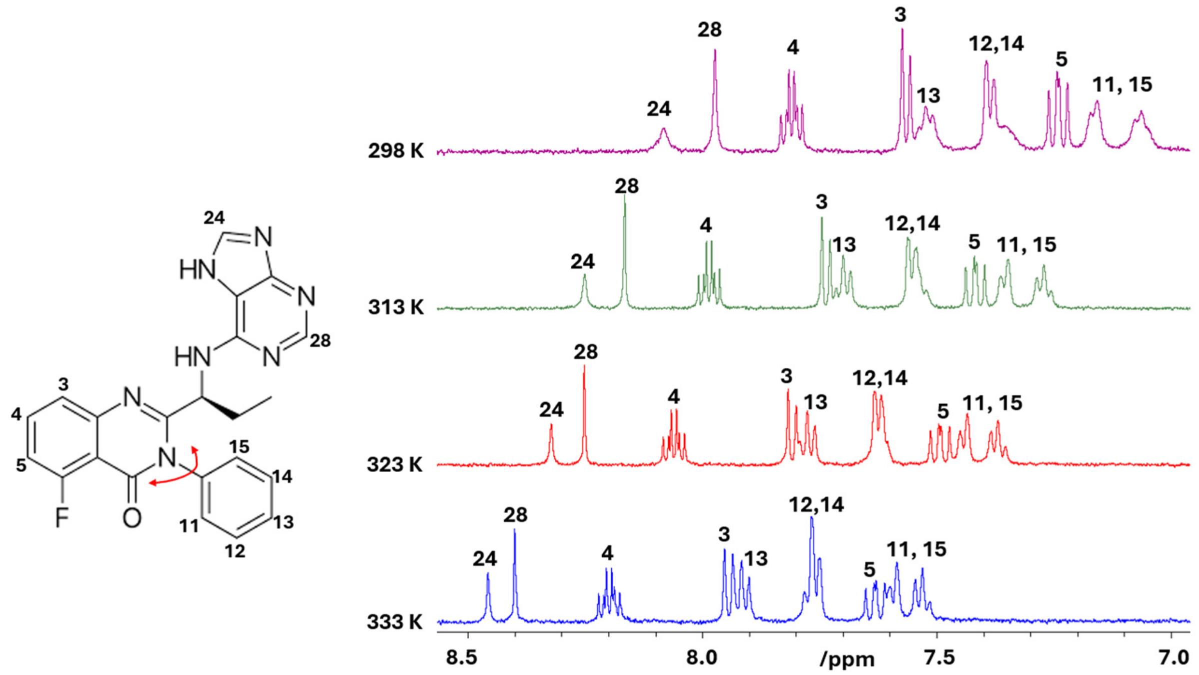

Figure S18. Chemical structure of *S*-IDE, with atom numbering and marketed the restricted rotation around the *C-N* bond. Representative ^{1}H NMR spectra of IDE recorded in D_2O (pD 3) at 500 MHz over the temperature range of 298-333 K, showing sharpening of aromatic signals upon heating due to faster rotamer exchange.

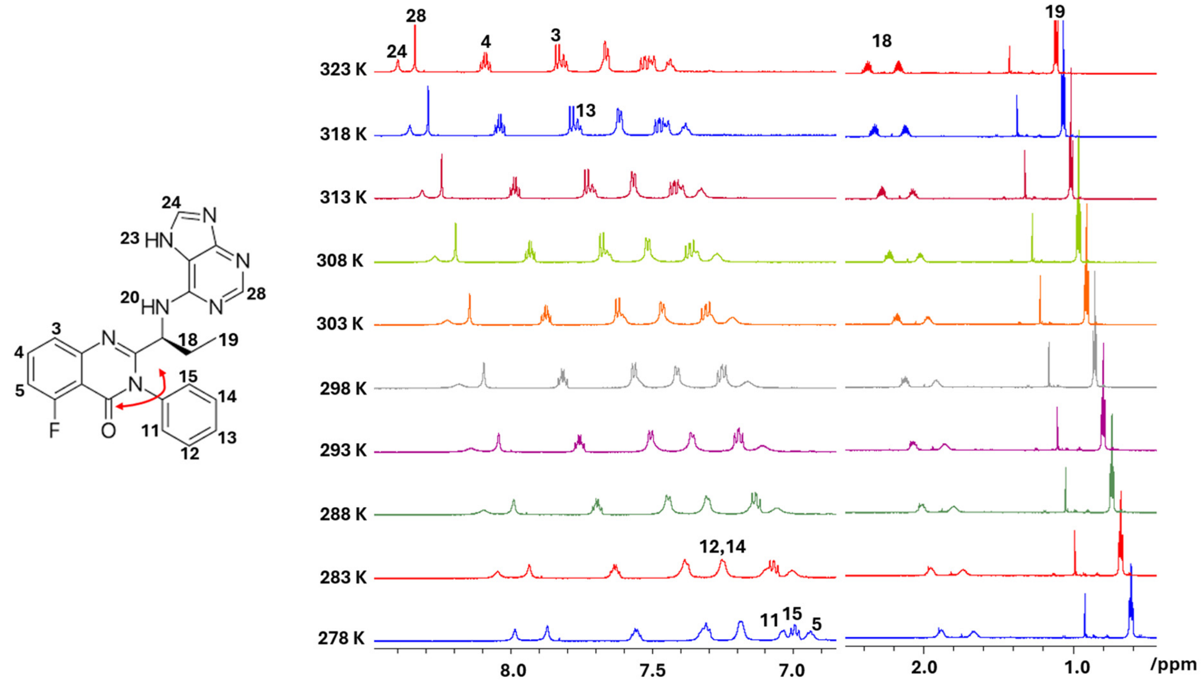

Figure S19. Chemical structure of *S*-IDE, with atom numbering and marketed the restricted rotation around the *C-N* bond. Representative ^{1}H NMR spectra of IDE recorded in D_2O (pD 3) at 700 MHz over the temperature range of 278-323 K.

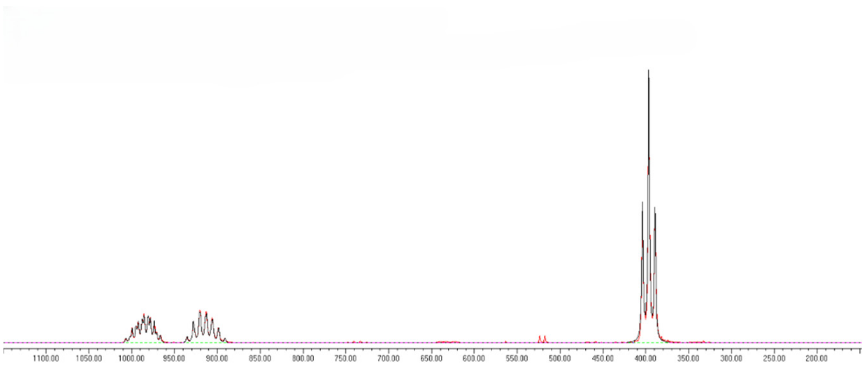

Figure S20. Overlay of the experimental (red) and simulated (black) ^{1}H NMR spectra of IDE recorded in DMSO-d_6 at 500 MHz and 363 K, showing the aliphatic region containing the H18 and H19 proton signals.

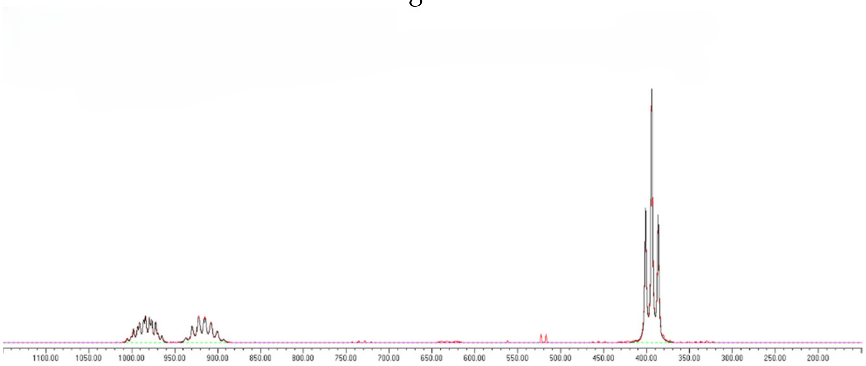

Figure S21. Overlay of the experimental (red) and simulated (black) ^{1}H NMR spectra of IDE recorded in DMSO-d_6 at 500 MHz and 353 K, showing the aliphatic region containing the H18 and H19 proton signals.

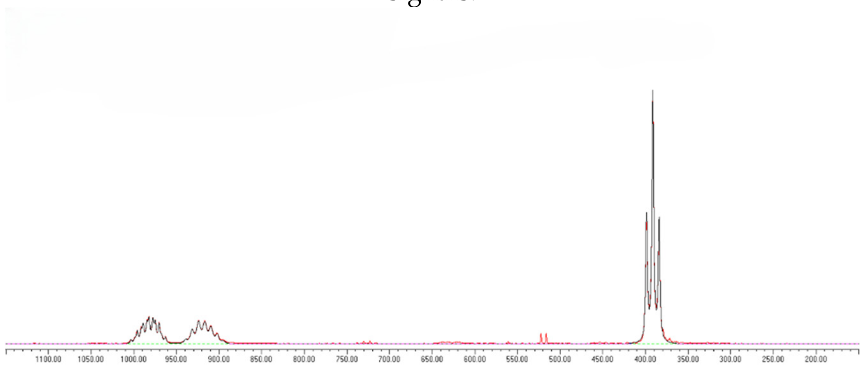

Figure S22. Overlay of the experimental (red) and simulated (black) ^{1}H NMR spectra of IDE recorded in DMSO-d_6 at 500 MHz and 343 K, showing the aliphatic region containing the H18 and H19 proton signals.

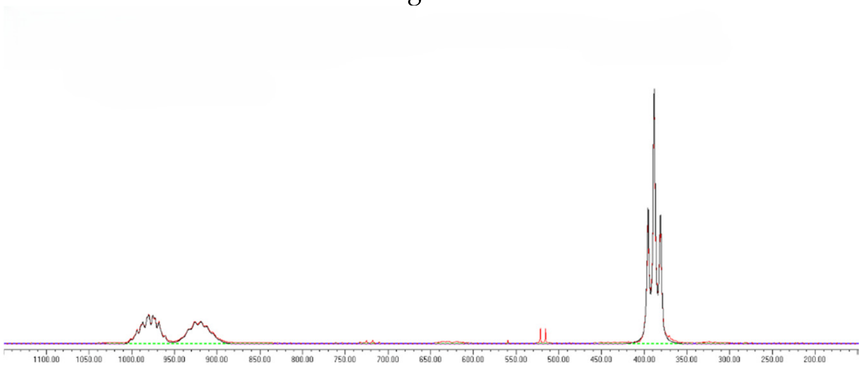

Figure S23. Overlay of the experimental (red) and simulated (black) ^{1}H NMR spectra of IDE recorded in DMSO-d_6 at 500 MHz and 333 K, showing the aliphatic region containing the H18 and H19 proton signals.

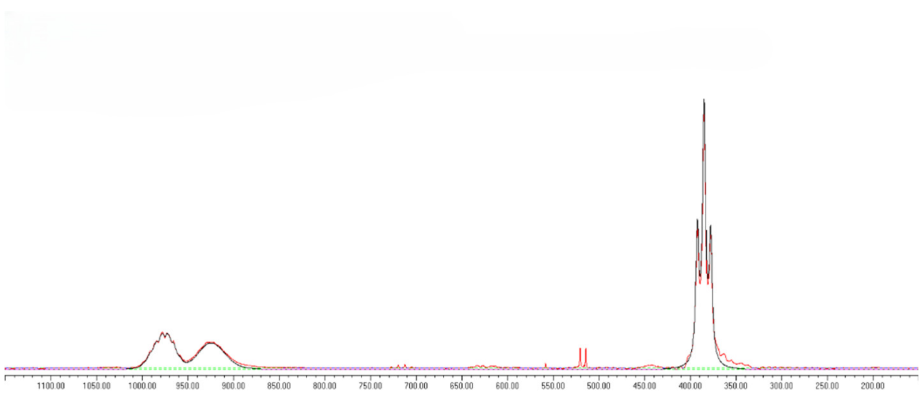

Figure S24. Overlay of the experimental (red) and simulated (black) ^{1}H NMR spectra of IDE recorded in DMSO-d_6 at 500 MHz and 323 K, showing the aliphatic region containing the H18 and H19 proton signals.

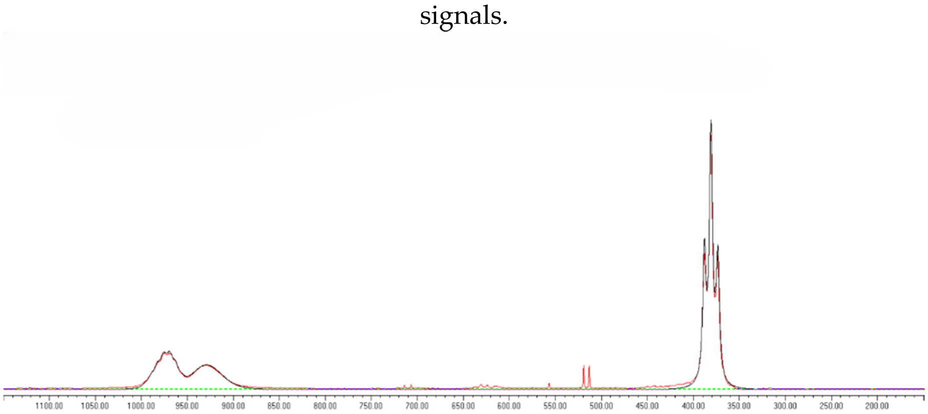

Figure S25. Overlay of the experimental (red) and simulated (black) ^{1}H NMR spectra of IDE recorded in DMSO-d_6 at 500 MHz and 313 K, showing the aliphatic region containing the H18 and H19 proton signals.

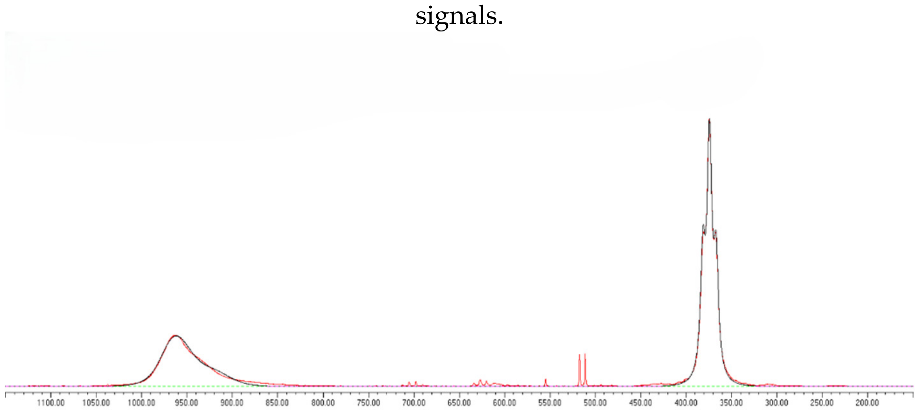

Figure S26. Overlay of the experimental (red) and simulated (black) ^{1}H NMR spectra of IDE recorded in DMSO-d_6 at 500 MHz and 298 K, showing the aliphatic region containing the H18 and H19 proton signals.

Table S13. ^1H assignment of idelalisib (*S*-IDE), (in D_2O, pD 3; 298 K; 500 MHz). For the atomic positions of *S*-IDE see Figure S8.

Atomic position	^1H δ (ppm)
1	-
2	-
3	7.54 (d, J=8.5 Hz; 1H)
4	7.79 (td, J= 8.5; 5.4 Hz; 1H)
5	7.22 (ddd; J=11.8; 8.5;0.7 Hz; 1H)
6	-
7	-
8	-
9	-
10	-
11	7.14 (m, broad, 1H)
12	7.35 (m, broad, 1H)
13	7.50 (m, broad, 1H)
14	7.35 (m, broad, 1H)
15	7.04 (m, broad, 1H)
16	-
17*	-
18a	1.88 (m, 2H)
18b	2.10 (m, 2H)
19	0.82 (t, 3H)
20	-
21	-
22	-
23	-
24	8.06 (s, broad, 1H)
25	-
26	-
27	-
28	7.95 (s, 1H)
29	-

* Not observed due to solvent suppression (5.36 ppm at 333 K).

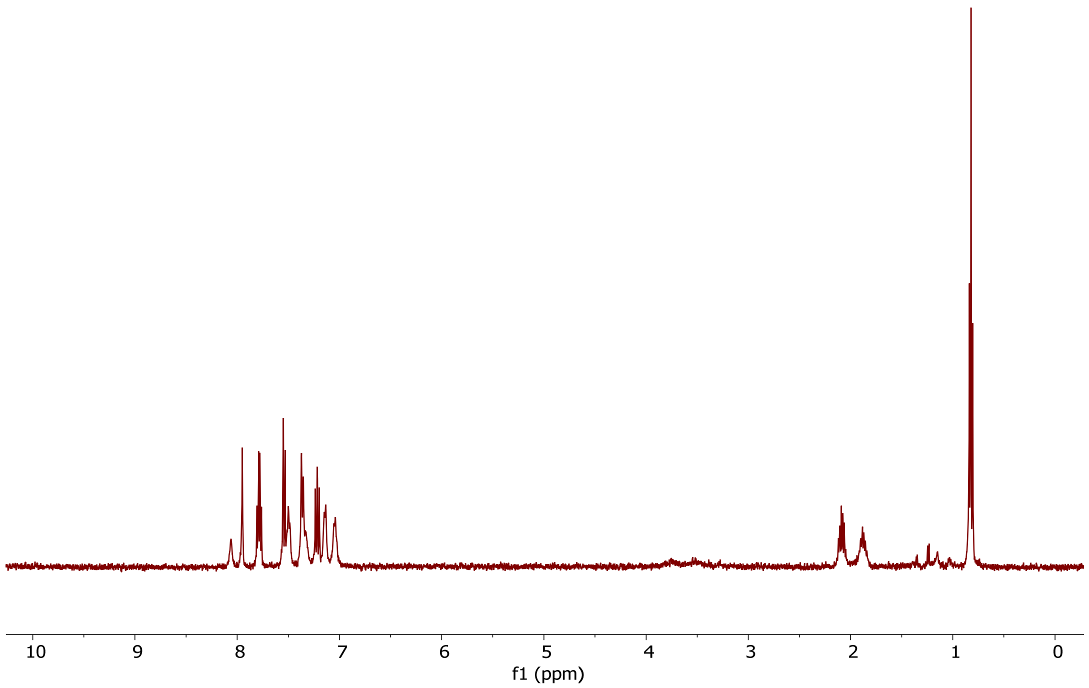

Figure S27. ^{1}H spectrum (water suppression) of idelalisib (*S*-IDE)**,** (in D_2O; pD 3; 298 K; 500 MHz).

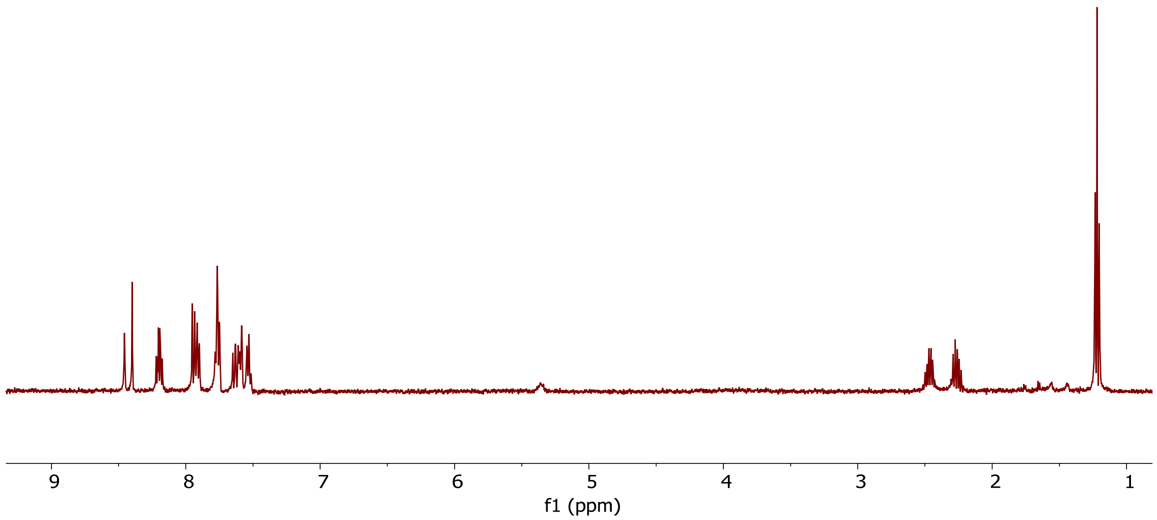

Figure S28. ^{1}H spectrum (water suppression) of idelalisib (*S*-IDE)**,** (in D_2O; pD 3; 333 K; 500 MHz).

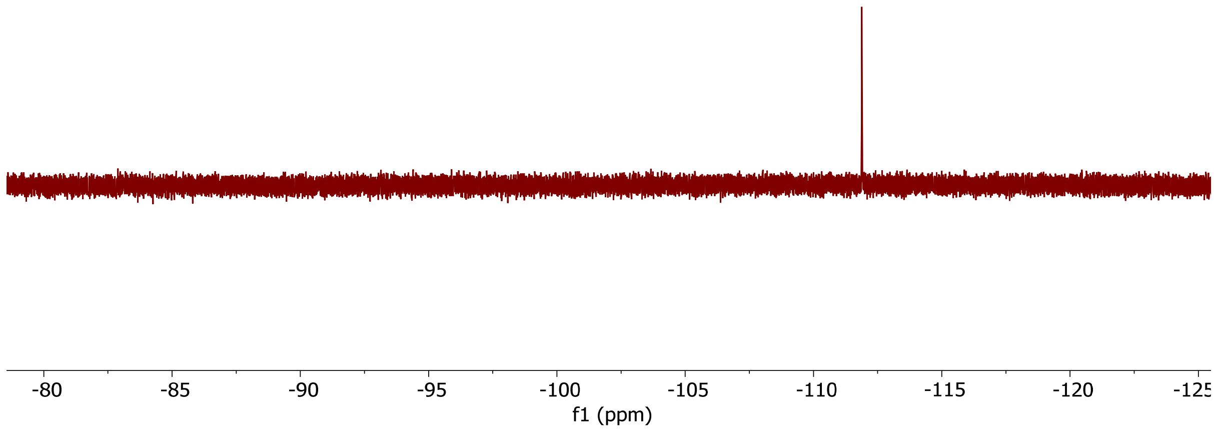

Figure S29. ^{19}F spectrum of R-idelalisib (*R*-IDE), (in D_2O; pD 3; 298 K; 376 MHz).

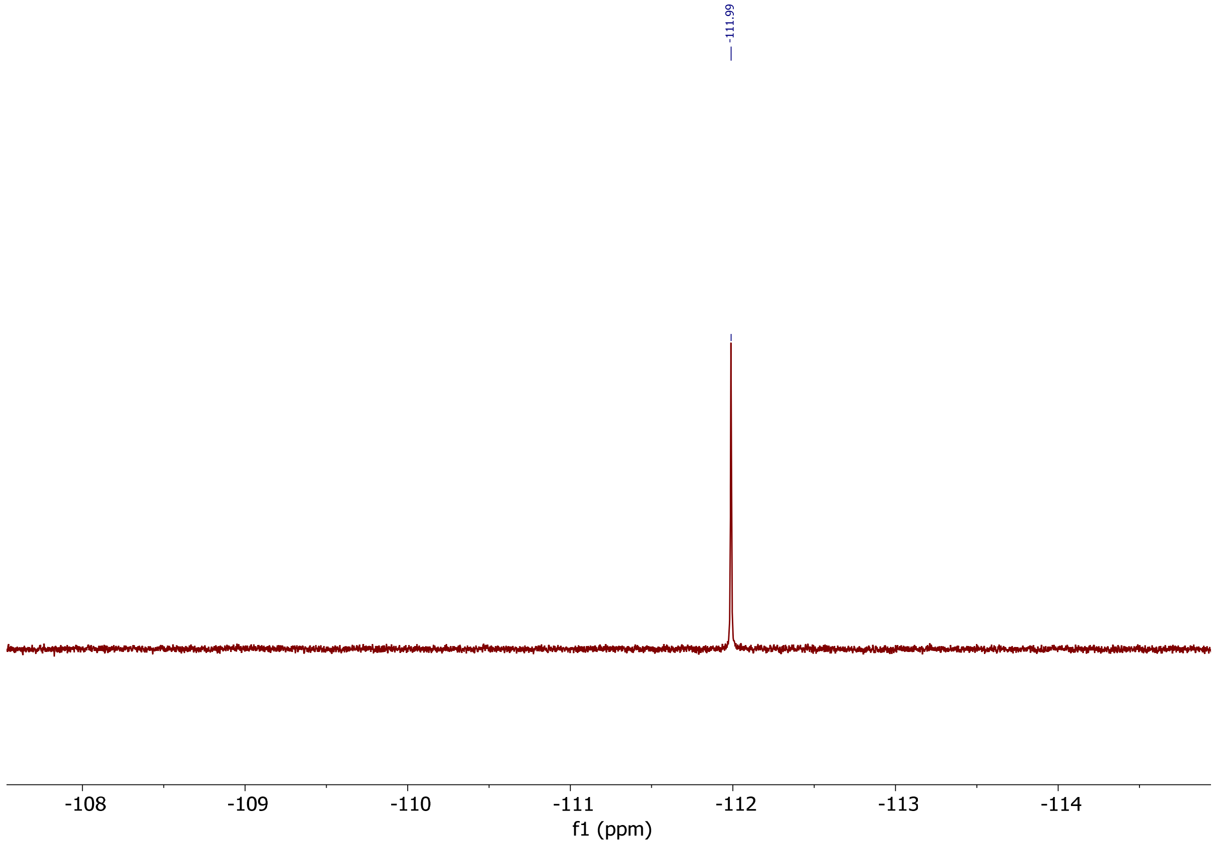

Figure S30. ^{19}F spectrum of idelalisib (*S*-IDE), (in D_2O; pD 3; 298 K; 376 MHz).

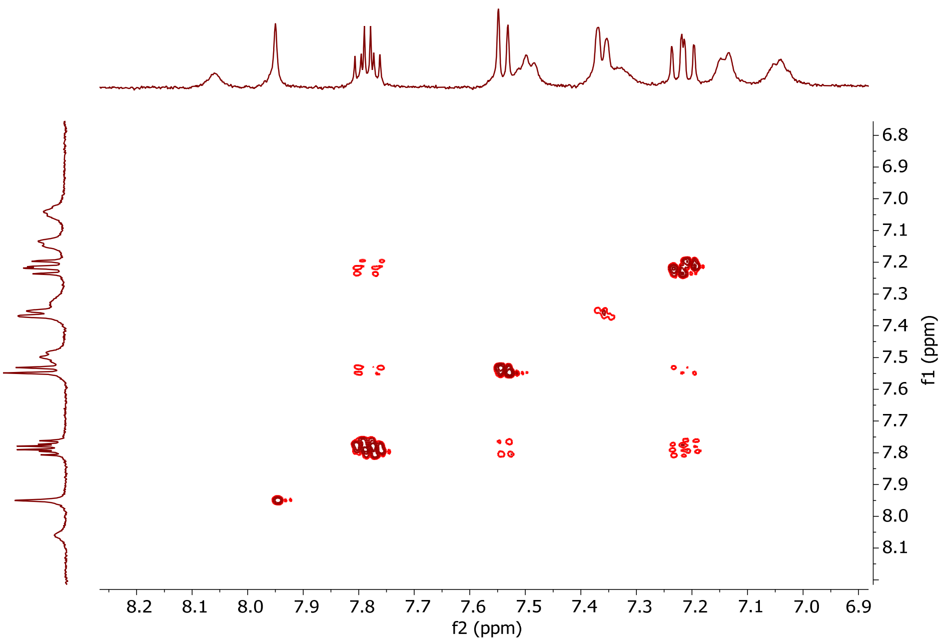

Figure S31. COSY spectrum of idelalisib (*S*-IDE), aromatic region (in D_2O; pD 3; 298 K; 500 MHz).

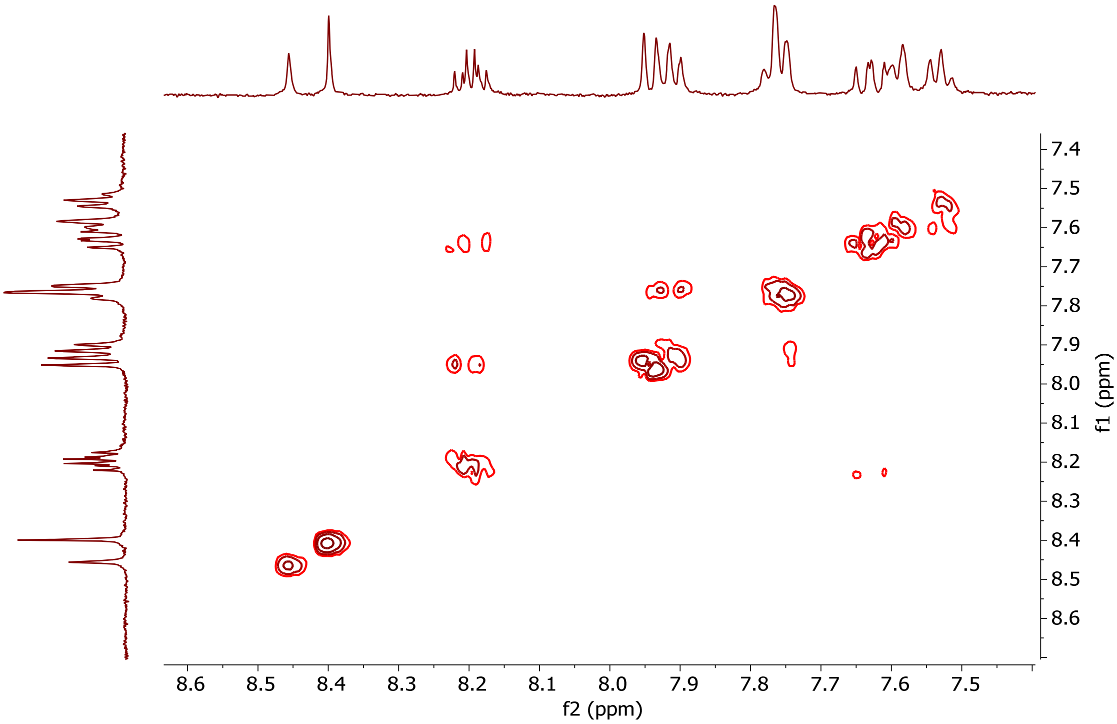

Figure S32. COSY spectrum of idelalisib (*S*-IDE), aromatic region (in D_2O; pD 3; 333 K; 500 MHz).

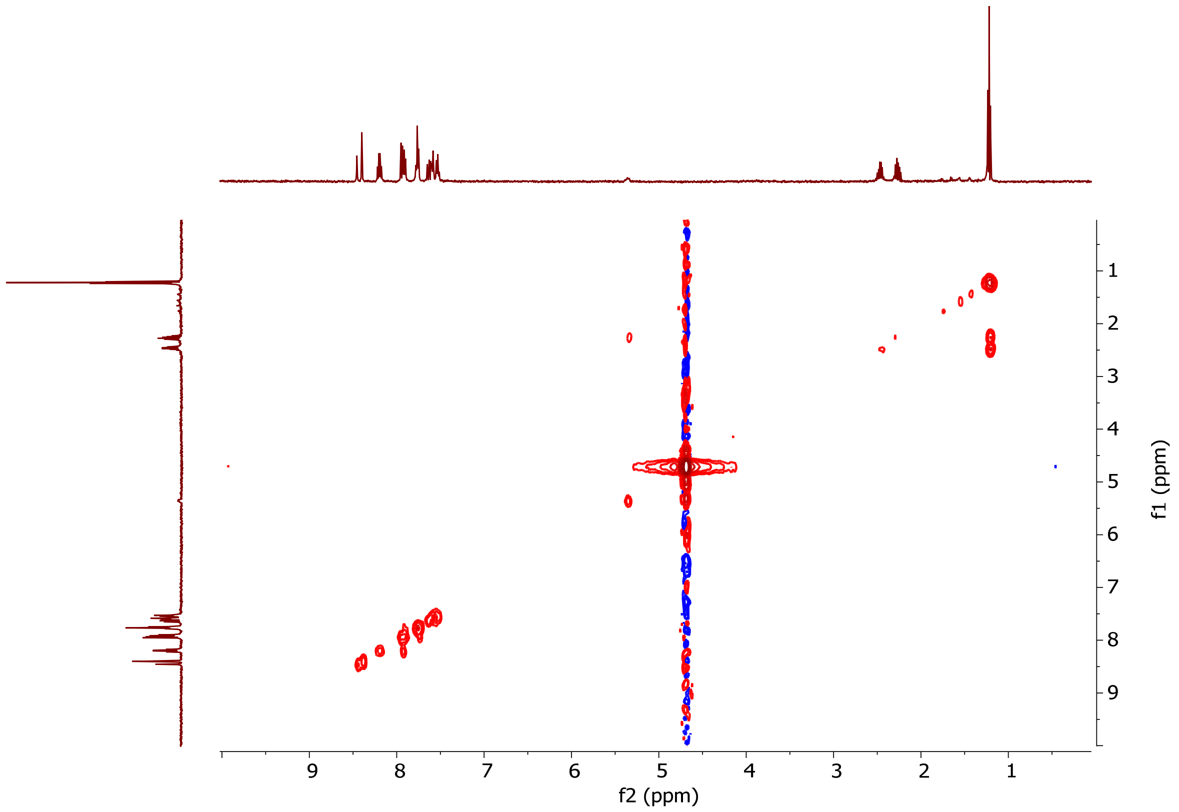

Figure S33. COSY spectrum of idelalisib (*S*-IDE)**,** (in D_2O; pD 3; 333 K; 500 MHz).

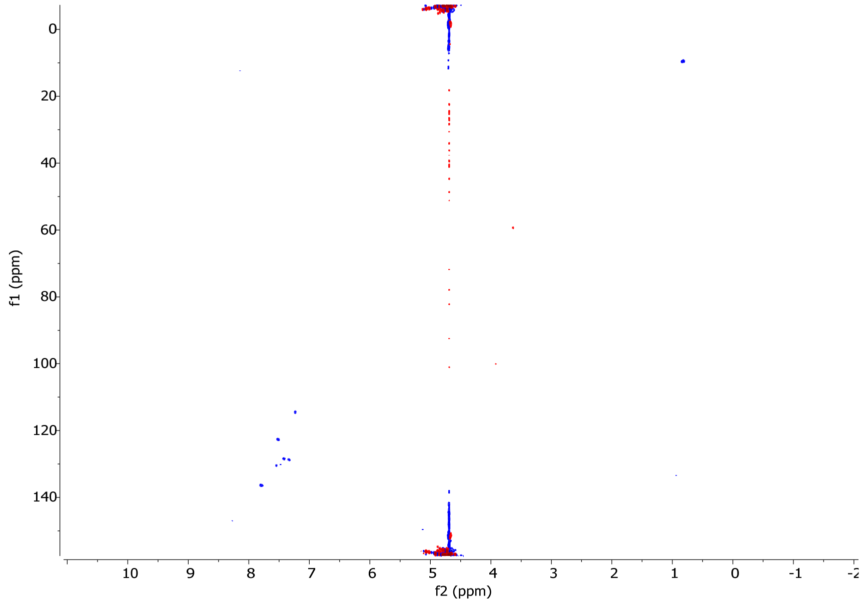

Figure S34. HSQC spectrum of *R*-idelalisib (*R*-IDE)**,** (in D_2O; pD 3; 298 K; 400 MHz).

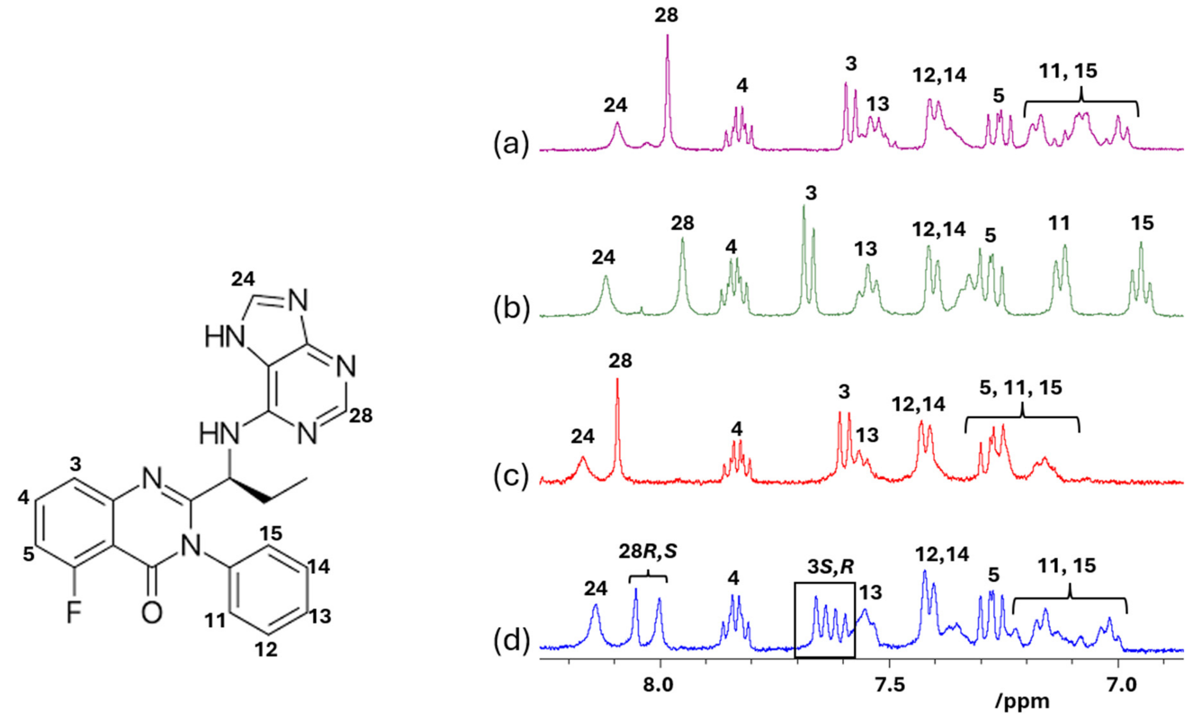

Figure S35. Chemical structure of *S*-IDE, with atom numbering (left). Representative ^{1}H spectra of (a) IDE in buffer, (b) *S*-IDE + β-CD (1:10), (c) *R*-IDE + β-CD (1:10), and (d) racemic IDE + β-CD (1:10). (D_2O; pD 3; 400 MHz, 298K).

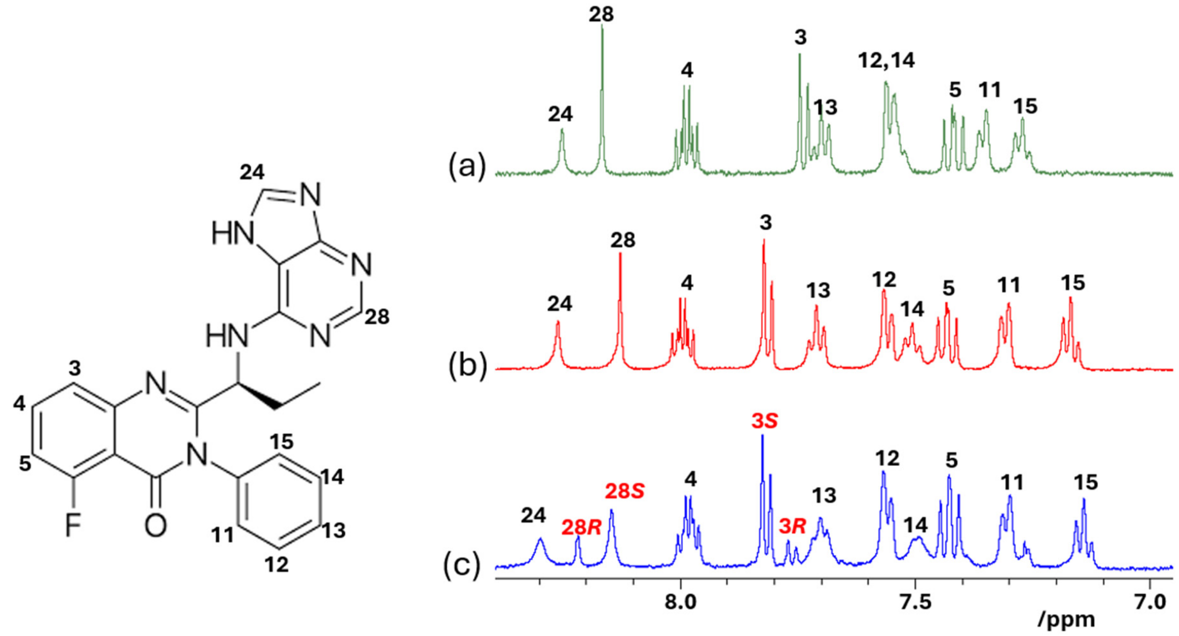

Figure S36. Chemical structure of *S*-IDE, with atom numbering (left). Representative ^{1}H spectra of (a) *S*-IDE in buffer, (b) *S*-IDE + β-CD (1:10), and (c) *R*-IDE + β-CD (1:10) spiked with *S*-IDE (*S*:*R* 4:1 ratio). (313K, D_2O, pD 3; 500 MHz).

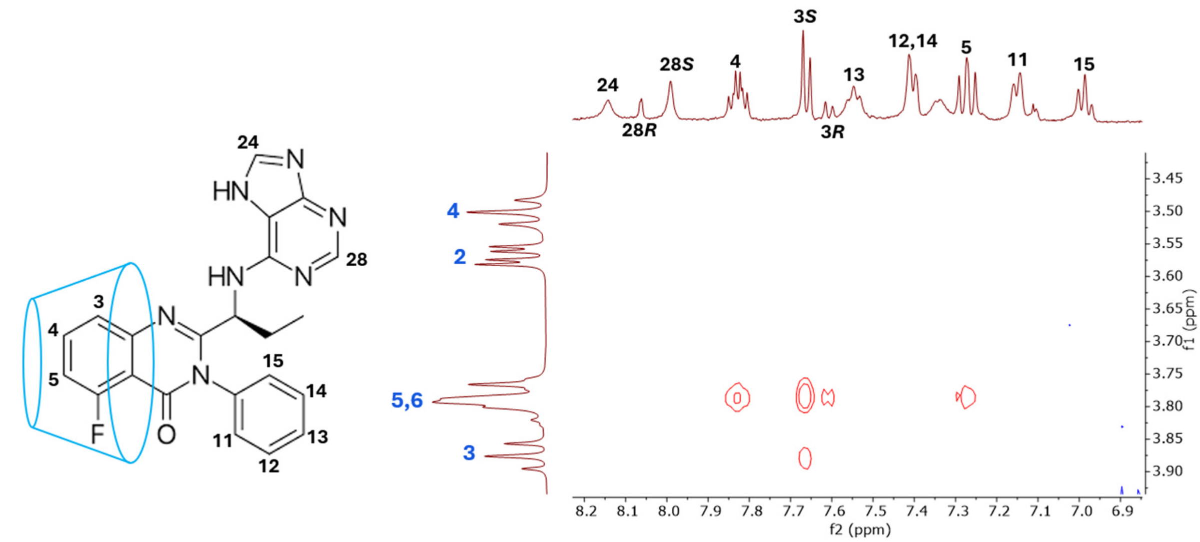

Figure S37. Chemical structure of *S*-IDE, with atom numbering and the β-CD-interacting moiety highlighted (left). Partial ROESY spectrum of *S*-IDE and β-CD spiked with *S*-IDE (right) (*S*:*R* 4:1 ratio; D_2O; pD 3; 500 MHz; 298 K).

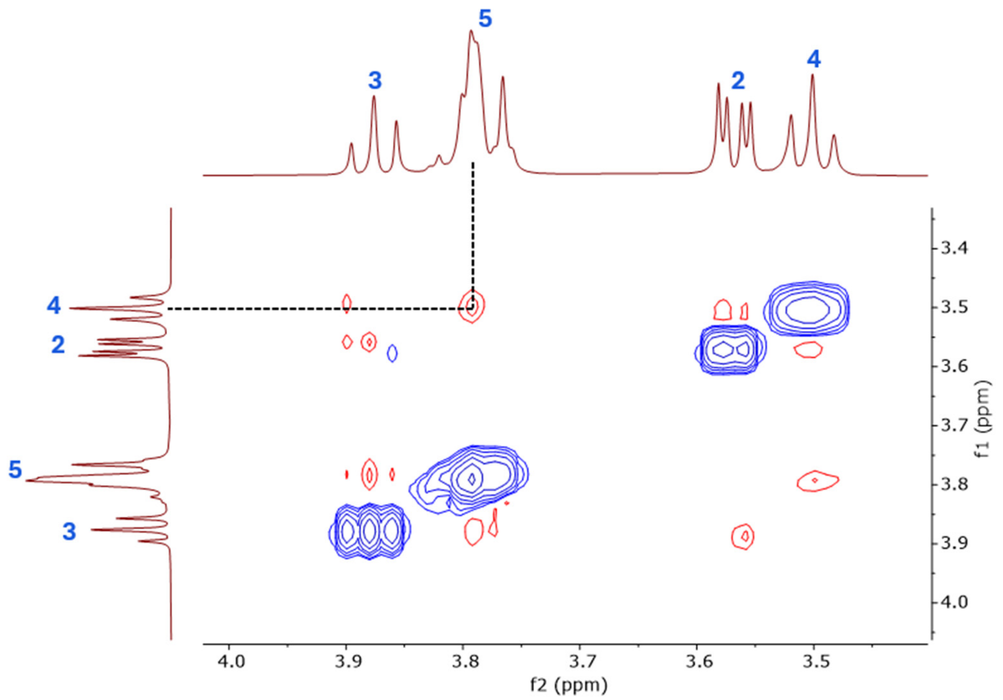

Figure S38. Partial ROESY spectrum of *S*-IDE - β-CD (1:1) complex (pD 3; 500 MHz; 298 K).

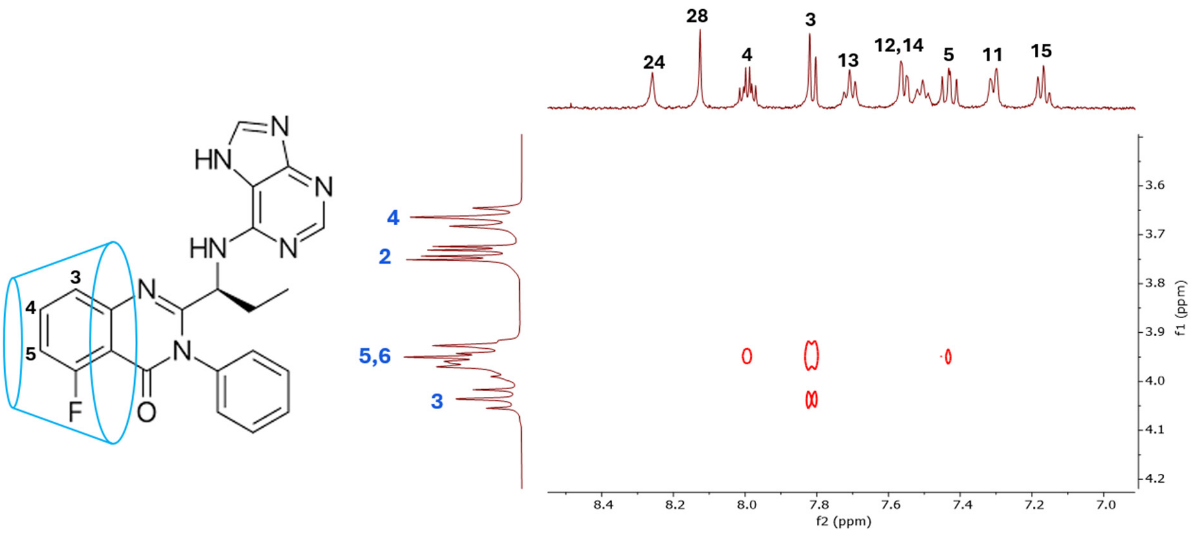

Figure S39. Chemical structure of *S*-IDE, with atom numbering and the β-CD-interacting moiety highlighted (left). Partial ROESY spectrum of IDE and β-CD (right) (1:1; pD 3; 500 MHz; 313 K).

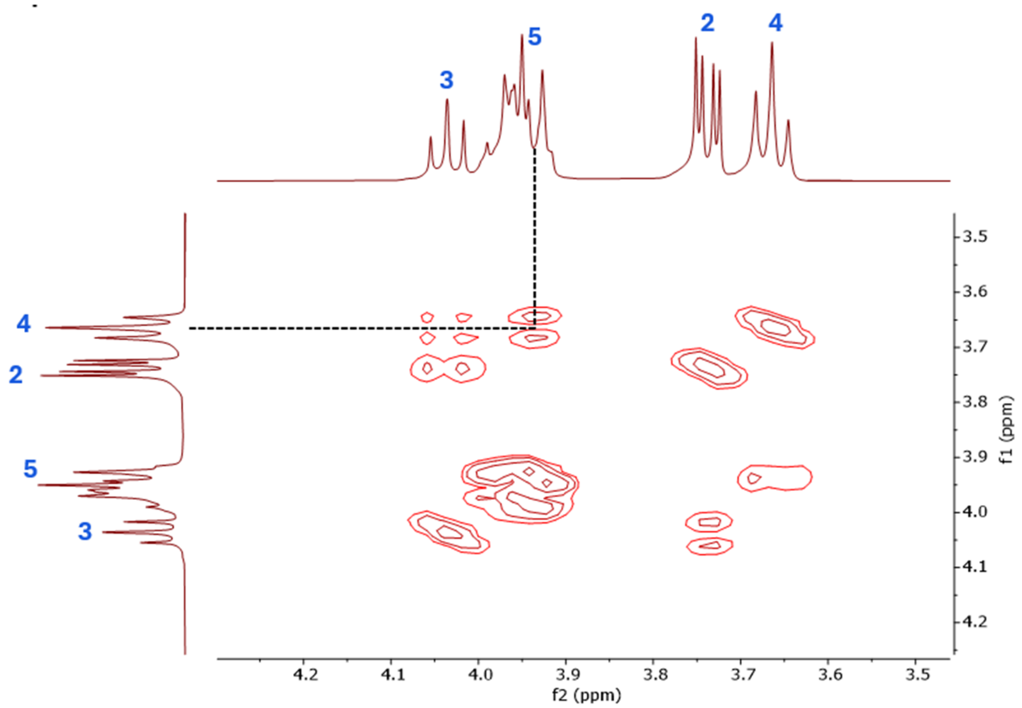

Figure S40. COSY spectrum of *S*-IDE - β-CD (1:1) complex; (D_2O; pD 3; 500 MHz; 313 K).

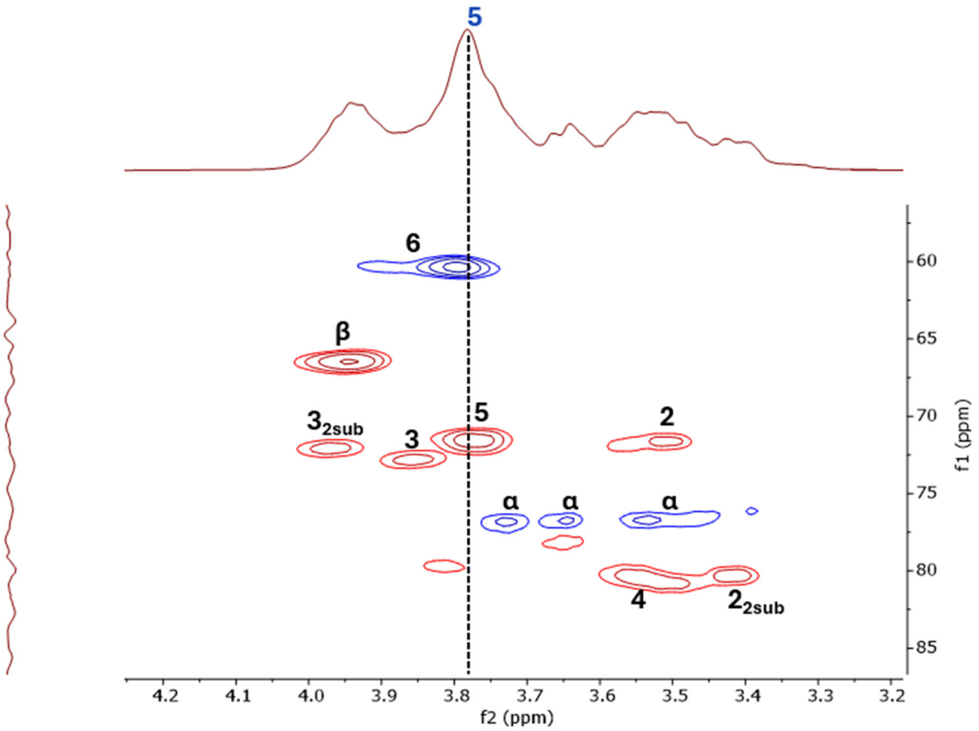

Figure S41. Partial DEPT-edited HSQC spectrum of *S*-IDE - HP-β-CD complex (1:10; pD 3; 400 MHz; 298 K).

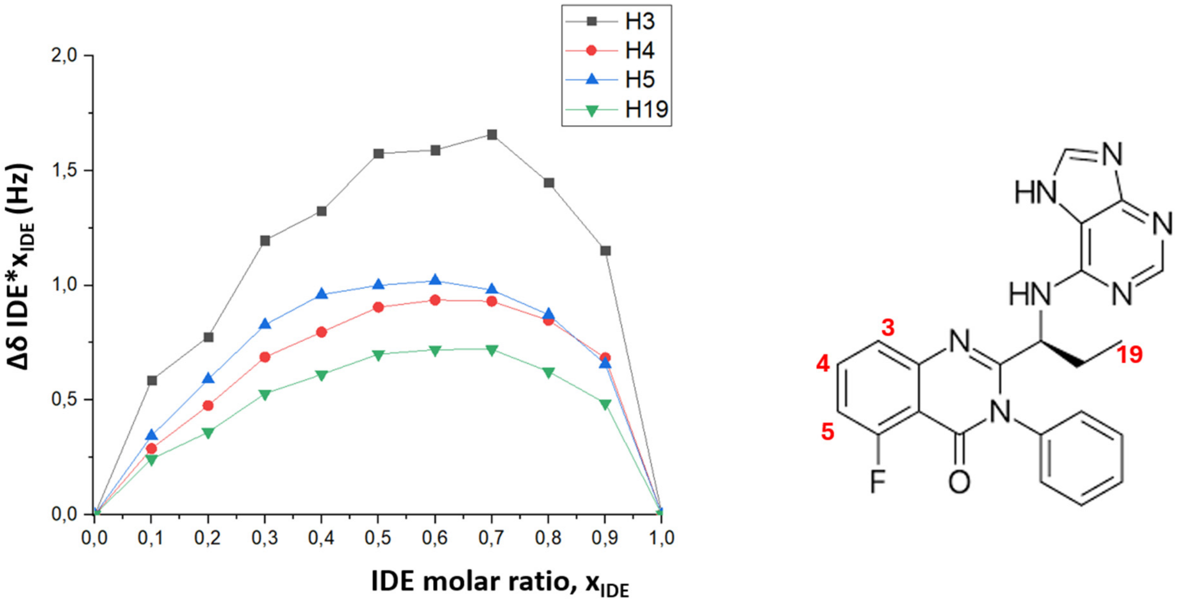

Figure S42. Job's plot of IDE and HP-β-CD (DS 6.8), (pD 3; 400 MHz; 298 K).

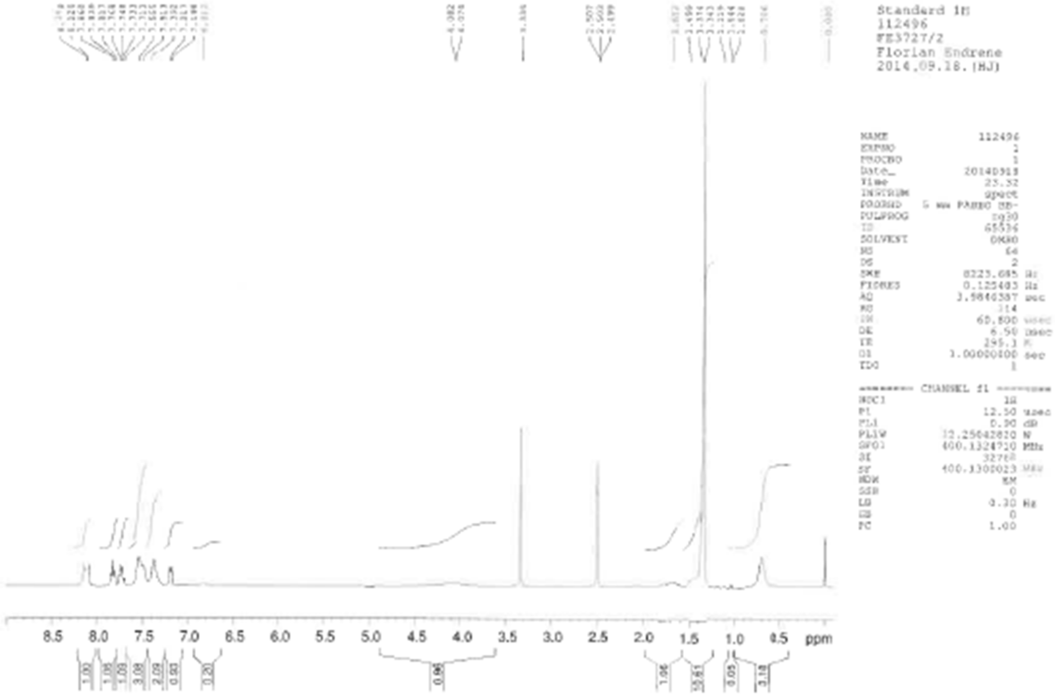

Figure S43. ^{1}H NMR spectrum of compound **4**.

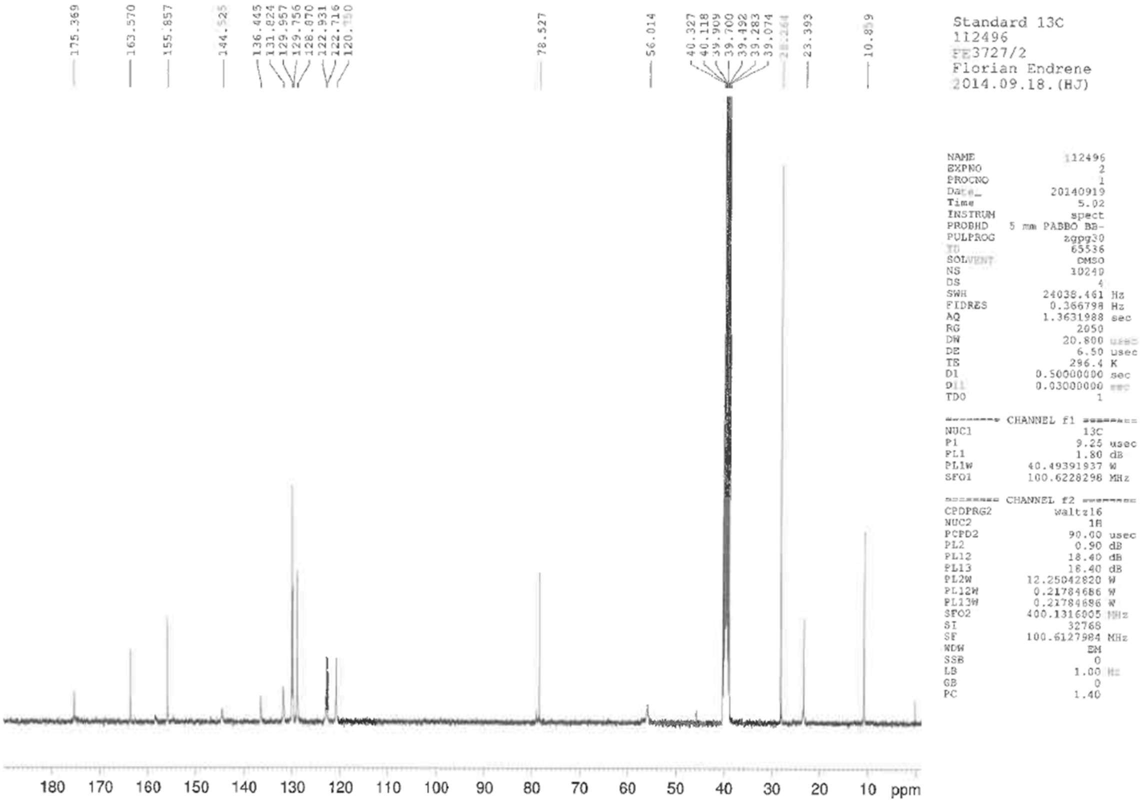

Figure S44. ^{13}C NMR spectrum of compound **4**.

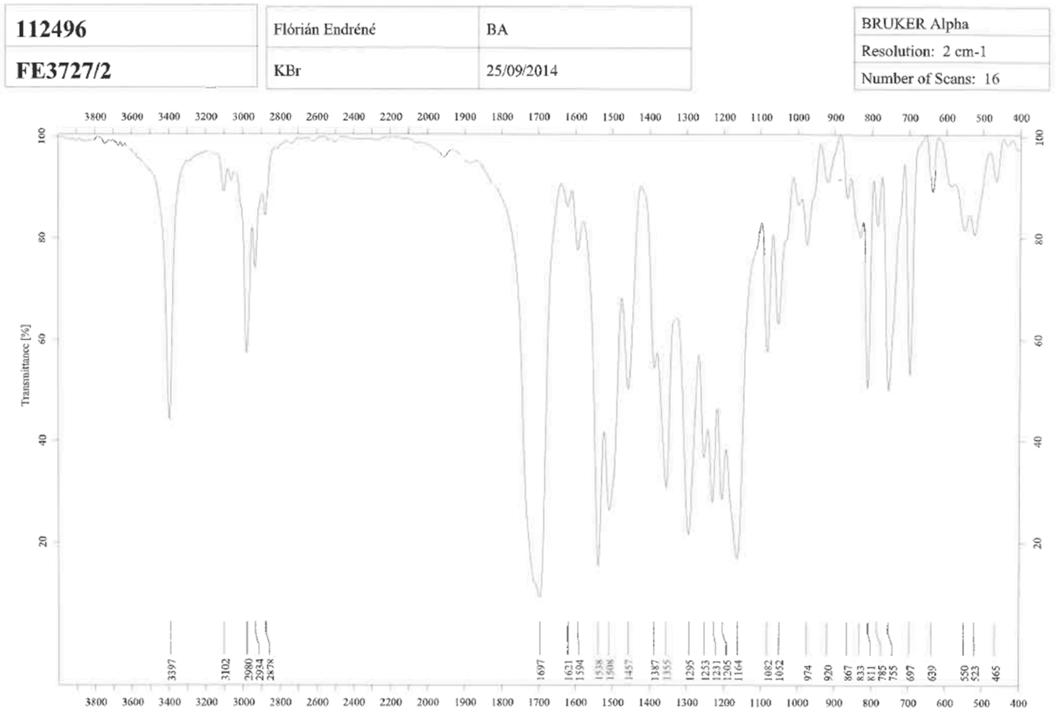

Figure S45. IR spectrum of compound **4**.

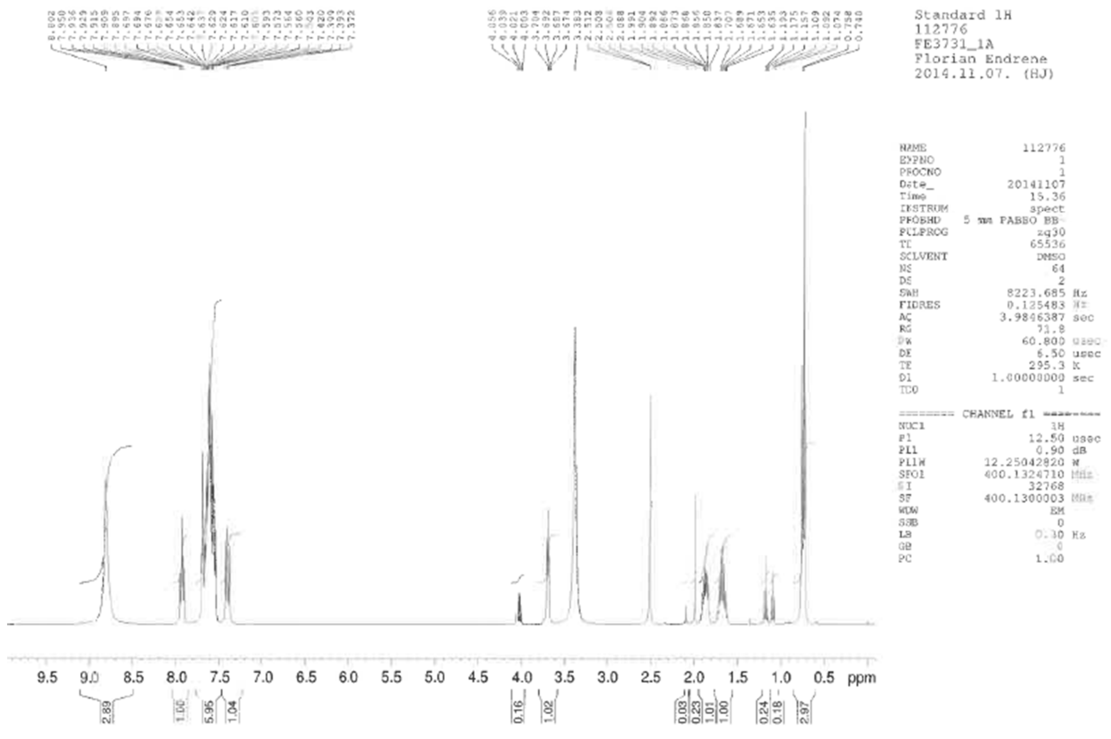

Figure S46. ^{1}H NMR spectrum of compound **6**.

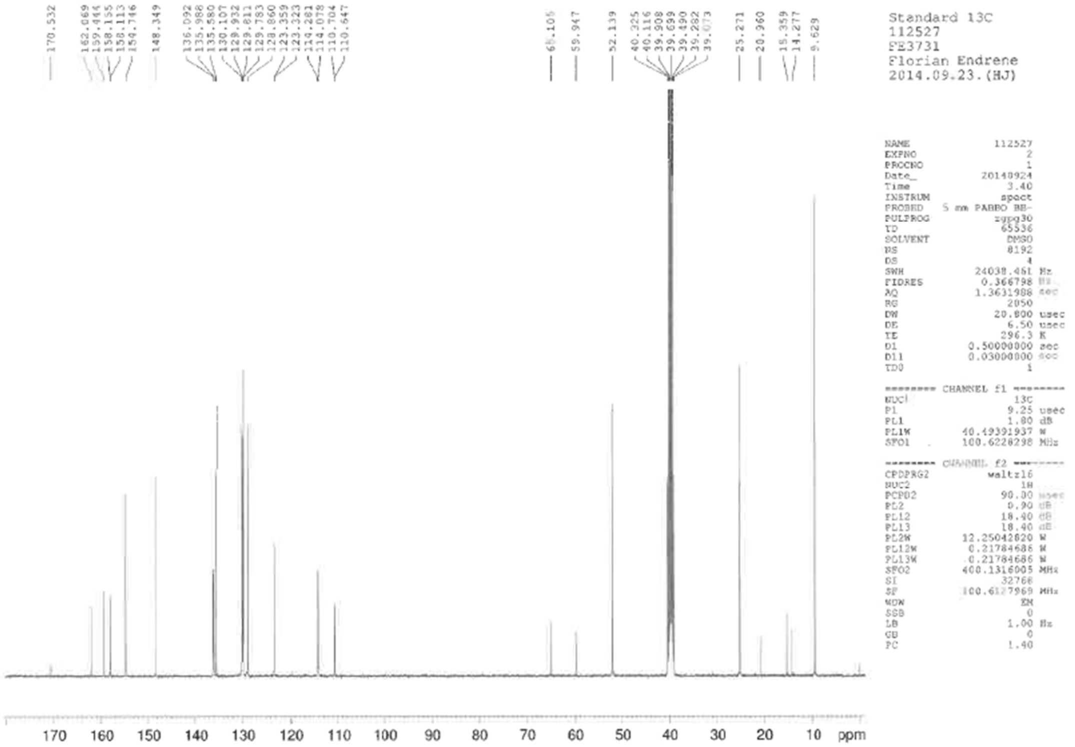

Figure S47. ^{13}C NMR spectrum of compound **6**.

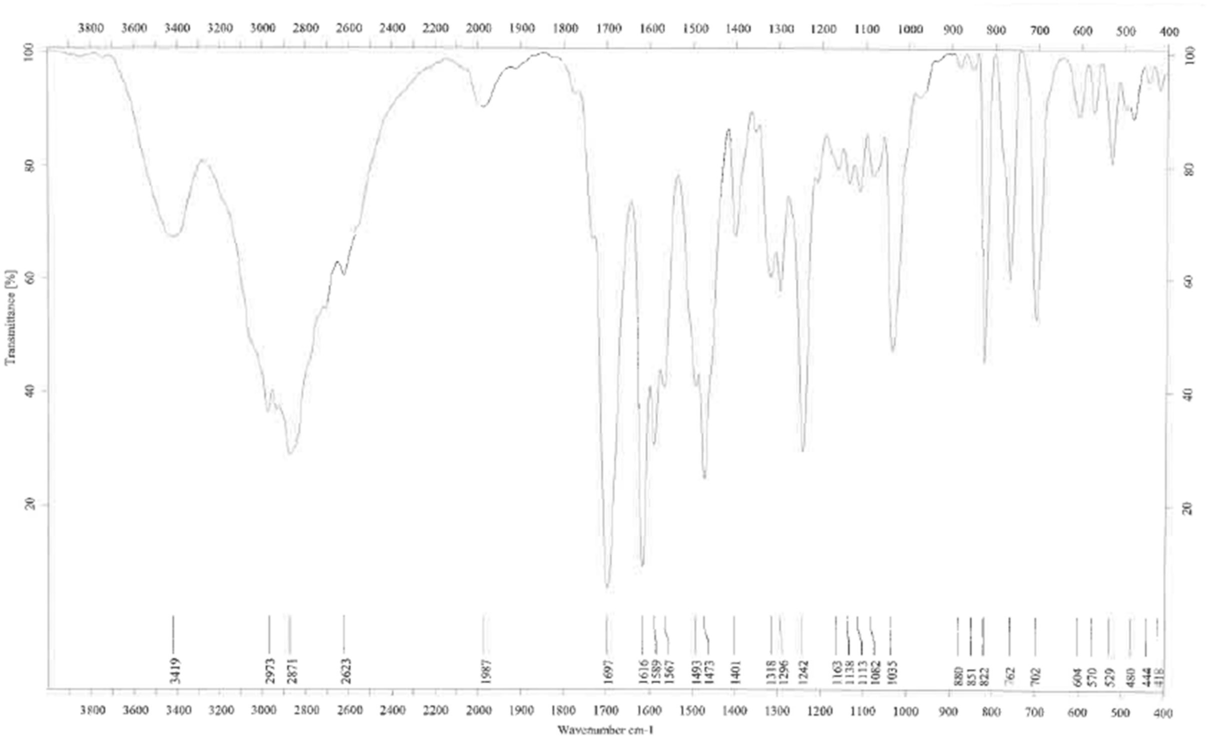

Figure S48. IR spectrum of compound **6**.

Table S14. The exchange rate constants (k_{exch}) determined by gNMR 5.0.6.0 softver, from the Variable Temperature NMR data recorded at 500 MHz in DMSO-d_6.

T / K	T^{-1} / K^{-1}	k_{exch} / s^{-1}	k / s^{-1}	ln (k / s^{-1})	ln [(k / T) / (s^{-1} · K^{-1})]
298	0.00336	54.61	109.2	4.693	–1.004
313	0.00319	135.0	270.0	5.598	–0.1478
323	0.00310	208.2	416.3	6.031	0.2538
333	0.00300	460.0	920.0	6.824	1.016
343	0.00292	797.3	1595	7.374	1.537
353	0.00283	1200	2400	7.783	1.917
363	0.00275	1700	3400	8.132	2.237
